# Chemodivergent photocatalytic access to 1-pyrrolines and 1-tetralones involving switchable C(sp^3^)–H functionalization

**DOI:** 10.3389/fchem.2022.1058596

**Published:** 2022-10-25

**Authors:** Shijing Tu, Zhongyu Qi, Weicai Li, Shiqi Zhang, Zhijie Zhang, Jun Wei, Lin Yang, Siping Wei, Xi Du, Dong Yi

**Affiliations:** ^1^ Central Nervous System Drug Key Laboratory of Sichuan Province, Department of Medicinal Chemistry, School of Pharmacy, Southwest Medical University, Luzhou, China; ^2^ Natural Products Research Center, Chengdu Institution of Biology, Chinese Academy of Science, Chengdu, Sichuan, China; ^3^ Department of Chemistry, School of Basic Medical Science, Southwest Medical University, Luzhou, China

**Keywords:** 1-pyrrolines, hydrogen atom transfer, [3+2] cyclization, [4+2] cyclization, 1-tetralones

## Abstract

A chemodivergent photocatalytic approach to 1-pyrrolines and 1-tetralones from alkyl bromides and vinyl azides has been developed through chemoselectively controllable intermolecular [3 + 2] and [4 + 2] cyclization. This photoredox-neutral two-component protocol involves intermolecular radical addition and switchable distal C(sp^3^)–H functionalization enabled by iminyl radical-mediated 1,5-hydrogen atom transfer. Meanwhile, chemoselectivity between C(sp^3^)–N bond formation and C(sp^3^)–C(sp^2^) bond formation is precisely switched by photocatalysts (Ru(bpy)_3_(PF_6_)_2_ vs. *fac*-Ir(ppy)_3_) and additives (base vs. acid).

## Introduction

**SCHEME 1:**
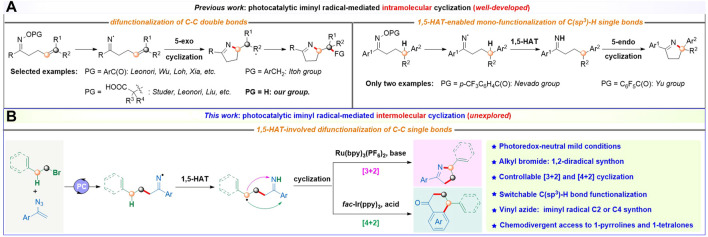
Photocatalytic access to 1-pyrrolines via iminyl radical-mediated cyclization.

1-Pyrroline motifs are core structures of a plethora of biologically active molecules such as natural products and drugs. Joined to this, they could also act as versatile synthetic intermediates of various valuable molecules including pyrrolidine alkaloids (Tyroller et al., 2002; Pluotno and Carmeli, 2005; Newcomb et al., 2016), ligands (Lasri et al., 2012), pharmaceuticals (Rosser and Faulkner, 1984; Schann et al., 2001; Tsukamoto et al., 2001; Aicher et al., 2011), etc (Miltyk and Pałka, 2000; Stapon et al., 2003). With the rapid development of visible-light-driven photoredox catalysis, photocatalytic iminyl radical-mediated cyclization reactions have emerged as greener and milder approaches for the straightforward synthesis of 1-pyrroline frameworks. One of the novel synthetic methods is photocatalytic iminyl radical-mediated intramolecular cyclization/functionalization of C–C double bonds for rapid synthesis of diversely functionalized 1-pyrroline derivatives developed by the groups of Leonori (Davies et al., 2015), Itoh (Usami et al., 2018), and Studer (Jiang and Studer, 2017), our group (Zhang et al., 2021), and others (Chen et al., 2020; Wang et al., 2020; Krylov et al., 2021) (Scheme 1A, left). On the other hand, photocatalytic iminyl radical-mediated intramolecular 1,5-hydrogen atom transfer (HAT)/cyclization cascade has been exploited by Nevado group and Yu group as a valuable tool for distal C(sp^3^)–H bond functionalization to access 1-pyrrolines and related scaffolds (Scheme 1A, right) (Shu and Nevado, 2017; Wu et al., 2021). Despite the marked advances achieved, these two iminyl radical-mediated approaches to 1-pyrroline skeletons are based on the self-elaboration of single puzzling oxime derivative. Thus, the development of novel, straightforward, and efficient approaches to construct 1-pyrroline architectures *via* photocatalytic iminyl radical-mediated intermolecular cyclization, especially with two or more simple and readily available starting materials, is highly desirable but challenging.

Meanwhile, vinyl azides, as another extremely attractive iminyl radical source, could provide iminyl radicals through the radical addition followed by the release of an N_2_ molecule to trigger further cyclization for the facile construction of various hetero- and carbocycles (Shu et al., 2017; Tang et al., 2017; Lei et al., 2018; Li et al., 2018; Tang et al., 2019; Liao et al., 2020; Lin et al., 2020; Jiao et al., 2021; Liang et al., 2021). In 2021, our group developed an unprecedented trifunctionalizing *ipso*-spirocyclization of vinyl azides with unactivated alkenes as 1,2,n-tri-radical precursors to afford novel spiroaminal skeletons through photocatalytic nitrogen radical-triggered cyclization-trapping-translocation-cyclization cascade (Qi et al., 2021). Prompted by this work and seminal pioneering reports on the unparalleled functional diversity of vinyl azides (Fu et al., 2017), we envisioned whether readily available alkyl bromides could be used as 1,2-diradical precursors and react with iminyl radical source vinyl azides to undergo the photocatalytic intermolecular radical addition, iminyl radical-mediated intramolecular 1,5-HAT (Dong et al., 2022), and sequential radical cyclization cascade, thus providing access to highly valuable 1-pyrroline architectures. Herein, we report an example of photocatalytic iminyl radical-mediated intermolecular [3 + 2] cyclization from alkyl bromides and vinyl azides to access structurally intriguing and highly functionalized five-membered heterocycle 1-pyrrolines (Scheme 1B). Interestingly, a chemodivergent approach to pharmaceutically important 1-tetralones (Taber et al., 2004; Odagi et al., 2017; Legoabe et al., 2018) (six-membered carbocycles) via photocatalytic iminyl radical-mediated intermolecular [4 + 2] cyclization from the same starting materials could also be established (Miller and Sarpong, 2011; Zhan et al., 2017; Najera et al., 2019).

## Results and discussion

To validate our hypothesis, diethyl 2-benzyl-2-bromomalonate **1a** and vinyl azide **2a** were firstly selected as model substrates to optimize the reaction conditions (Table 1). It was pleasing to find that when using *fac*-Ir(ppy)_3_ as the photocatalyst, K_2_CO_3_ as the base, and anhydrous CH_2_Cl_2_ as the solvent, the expected [3 + 2] cyclization product 1-pyrroline **3aa** could be obtained in 12% yield as well as the [4 + 2] cyclization product 1-tetralone **4aa** in relatively higher yield (entry 1). Subsequently, screening of photocatalysts demonstrated that replacing *fac*-Ir(ppy)_3_ with Ru(bpy)_3_(PF_6_)_2_ remarkably enhanced the reaction efficiency of [3 + 2] cyclization, whereas only a trace amount of the [4 + 2] cyclization product **4aa** was detected (entries 2 and 3). After selecting Ru(bpy)_3_(PF_6_)_2_ as an optimal photocatalyst, we further explored the effect of solvents and additives. However, when other solvents such as DMF, THF, and MeCN were tested, it led to the expected product **3aa** in the decreased yields of 13%, 46%, and 55%, respectively (entries 4–6). Furthermore, other tested bases delivered no more significant improvement, and notably, the yield of the desired product **3aa** dropped sharply to 12% without the addition of a base (entries 7–10), indicating that a base is critical to the success of this [3 + 2] cyclization. Interestingly, the yield of the desired product **3aa** could be further improved to 82% yield by adjusting the loading of reaction substrates (entries 11 and 12). As anticipated, control experiments indicated that visible light and photocatalyst *fac*-Ir(ppy)_3_ were indispensable for such photocatalytic [3 + 2] cyclization (entries 13 and 14). Delightfully, using AcOH instead of the essential base in the predominant [3 + 2] cyclization could not only enable the preferential [4 + 2] cyclization, but the yield of 1-tetralone **4aa** could also be improved from 60% to 76% by further increasing the amount of solvent CH_2_Cl_2_ (entries 15 and 16 vs. entry 1).

**TABLE 1:** Optimization of the reaction conditions^a^ ^,^ ^b^.

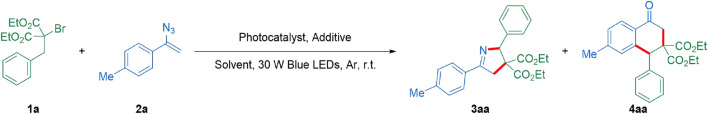					
Entry	Photocatalyst	Solvent	Additive	3aa Yield (%)	4aa Yield (%)
1	*f*ac-Ir(ppy)_3_	CH_2_Cl_2_	K_2_CO_3_	12	36
2	Ir(*p*-F-ppy)_3_	CH_2_Cl_2_	K_2_CO_3_	6	14
3	Ru(bpy)_3_(PF_6_)_2_	CH_2_Cl_2_	K_2_CO_3_	77	trace
4	Ru(bpy)_3_(PF_6_)_2_	DMF	K_2_CO_3_	13	n.d.
5	Ru(bpy)_3_(PF_6_)_2_	THF	K_2_CO_3_	46	n.d.
6	Ru(bpy)_3_(PF_6_)_2_	MeCN	K_2_CO_3_	55	n.d.
7	Ru(bpy)_3_(PF_6_)_2_	CH_2_Cl_2_	K_3_PO_4_	66	n.d.
8	Ru(bpy)_3_(PF_6_)_2_	CH_2_Cl_2_	2,6-lutidine	72	n.d.
9	Ru(bpy)_3_(PF_6_)_2_	CH_2_Cl_2_	DABCO	70	n.d.
10	Ru(bpy)_3_(PF_6_)_2_	CH_2_Cl_2_	—	12	16
11^c^	Ru(bpy)_3_(PF_6_)_2_	CH_2_Cl_2_	K_2_CO_3_	64	trace
12^d^	Ru(bpy)_3_(PF_6_)_2_	CH_2_Cl_2_	K_2_CO_3_	**82**	8
13	—	CH_2_Cl_2_	K_2_CO_3_	n.d.	n.d.
14^e^	Ru(bpy)_3_(PF_6_)_2_	CH_2_Cl_2_	K_2_CO_3_	n.d.	n.d.
15^f^	*fac*-Ir(ppy)_3_	CH_2_Cl_2_	AcOH	Trace	60
16^f^ ^,^ ^g^	*fac*-Ir(ppy)_3_	CH_2_Cl_2_	AcOH	10	**76**

^a^ Reaction conditions: **1a** (0.1 mmol), **2a** (0.2 mmol), photocatalyst (2 mol%), solvent (1 ml), additive (0.15 mmol), 30 W blue LEDs, argon atmosphere, r.t., 9 h. n.d. = not detected.

^b^ Yields were determined by ^1^H NMR using dibromomethane as an internal standard.

^c^ **2a** (0.15 mmol).

^d^ **2a** (0.25 mmol).

^e^ Without LEDs.

^f^ Additive (0.1 mmol).

^g^ Solvent (2 ml).

**SCHEME 2:**
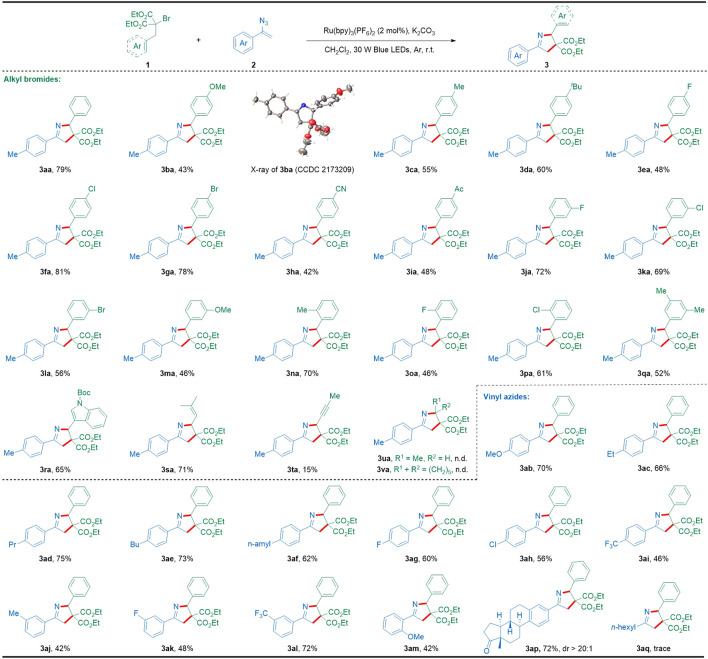
Substrate scope with respect to [3 + 2] cyclization. Reaction conditions: **1** (0.2 mmol), **2** (0.5 mmol), Ru(bpy)_3_(PF_6_)_2_ (2 mol%), K_2_CO_3_ (0.3 mmol), CH_2_Cl_2_ (2 ml), 30 W blue LEDs, argon atmosphere, r.t., 9 h, in a sealed tube; isolated yields based on **1** after the chromatographic purification.

Having established the optimal reaction conditions (Table 1, entry 12), we next explored the substrate scope of alkyl bromides and vinyl azides to extend the synthetic potential and generality of this method as summarized in Scheme 2. Firstly, the generality of this photocatalytic [3 + 2] cyclization with regard to alkyl bromides was examined. Generally, a diverse array of alkyl bromides bearing different electronic groups at various positions of the phenyl moiety could be successfully transformed into the desired 1-pyrroline products (**3aa**–**3qa**). Notably, the molecular structure of the representative 1-pyrroline **3ba** was confirmed by X-ray crystallography (CCDC 2173209). Gratifyingly, this photocatalytic cyclization tolerated well a broad range of diverse functionalities such as alkyl (**3ca**, **3da**, **3na**, and **3qa**), alkoxyl (**3ba** and **3ma**), cyano (**3ha**), and acetyl (**3ia**), as well as halogen (**3ea**–**3ga**, **3ja**–**3la**, and **3oa**–**3pa**) that could provide opportunities for further product functionalization. Interestingly, the [3 + 2] cyclization reaction between indole-derived bromide **1r** and vinyl azide **2a** also proceeded smoothly to produce the corresponding 1-pyrroline **3ra** with a satisfactory yield. Additionally, homoallylic and homopropargyl-substituted bromides have also been proven to be suitable substrates for this [3 + 2] cyclization, providing the desired highly substituted 1-pyrrolines (**3sa** and **3ta**) with yields of 71% and 15%, while saturated alkyl bromides (**1u** and **1v**) failed to give the desired products. Subsequently, we examined the scope of vinyl azides with 2-benzyl-2-bromomalonate **1a** as the coupling partner. Different substituents on the aromatic ring of the vinyl azides were found compatible with the [3 + 2] cyclization conditions to obtain the desired products in moderate to good yields (**3ab**–**3am**). It is worth noting that the estrone-derived vinyl azide **2p** effectively participated in such [3 + 2] cyclization to give the structurally intriguing 1-pyrroline derivative **3ap**, demonstrating the capacity of this protocol in late-stage modification of complex bioactive molecules. However, alkyl-substituted vinyl azide seemed to be not suitable for this cyclization and provided the unseparated mixture including a trace amount of the expected product **3aq**.

**SCHEME 3:**
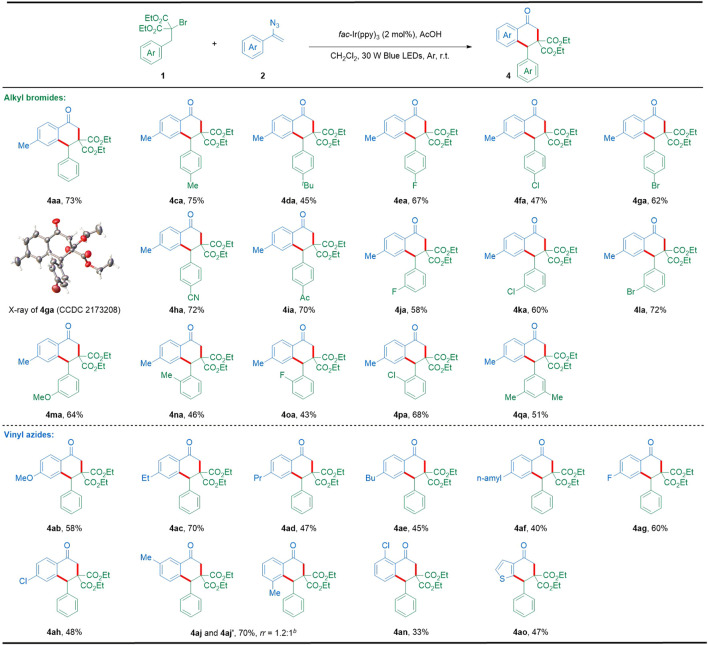
Substrate scope with respect to [4 + 2] cyclization. Reaction conditions: **1** (0.2 mmol), **2** (0.4 mmol), *fac*-Ir(ppy)_3_ (2 mol%), AcOH (0.2 mmol), CH_2_Cl_2_ (4 ml), 30 W blue LEDs, argon atmosphere, r.t., 9 h, in a sealed tube; isolated yields based on **1** after the chromatographic purification. *rr*: regioisomeric ratio.

After completing the [3 + 2] cyclization, we continued to expand the substrate scope to afford the [4 + 2] cyclization product 1-tetralones under the conditions of *fac*-Ir(ppy)_3_ as the photocatalyst and AcOH as the additive (Table 1, entry 16). As illustrated in Scheme 3, this [4 + 2] cyclization showed good tolerance of various substituents such as alkyl, halogen, cyano, acetyl, and alkoxyl, providing a great variety of highly functionalized 1-tetralones (**4aa**–**4qa**). In particular, the molecular structure of the representative 1-tetralone **4ga** was also confirmed by X-ray crystallographic analysis (CCDC 2173208). On the other hand, a panel of vinyl azides with electron-donating or electron-withdrawing groups at the para-position of the aromatic ring could be successfully converted into the corresponding 1-tetralones (**4ab**–**4ah**) in satisfactory yields. However, vinyl azide with methyl at the meta-position of the aromatic ring furnished the corresponding 1-tetralones as mixtures of regio-isomer (**4aj** and **4aj′**). In addition, the ortho-substituted vinyl azide reacted smoothly with **1a** to give the corresponding 1-tetralone in an acceptable yield (**4an**). Furthermore, this photocatalytic [4 + 2] cyclization also displayed good tolerance of other important heteroaromatics including thiophene (**4ao**). However, during our preparation of this manuscript, Xu, Hu, and co-workers also reported an elegant radical cascade cyclization between vinyl azides and alkyl bromides toward 1-tetralone skeletons by using a metal-free photocatalyst (Jiao et al., 2021).

**SCHEME 4:**
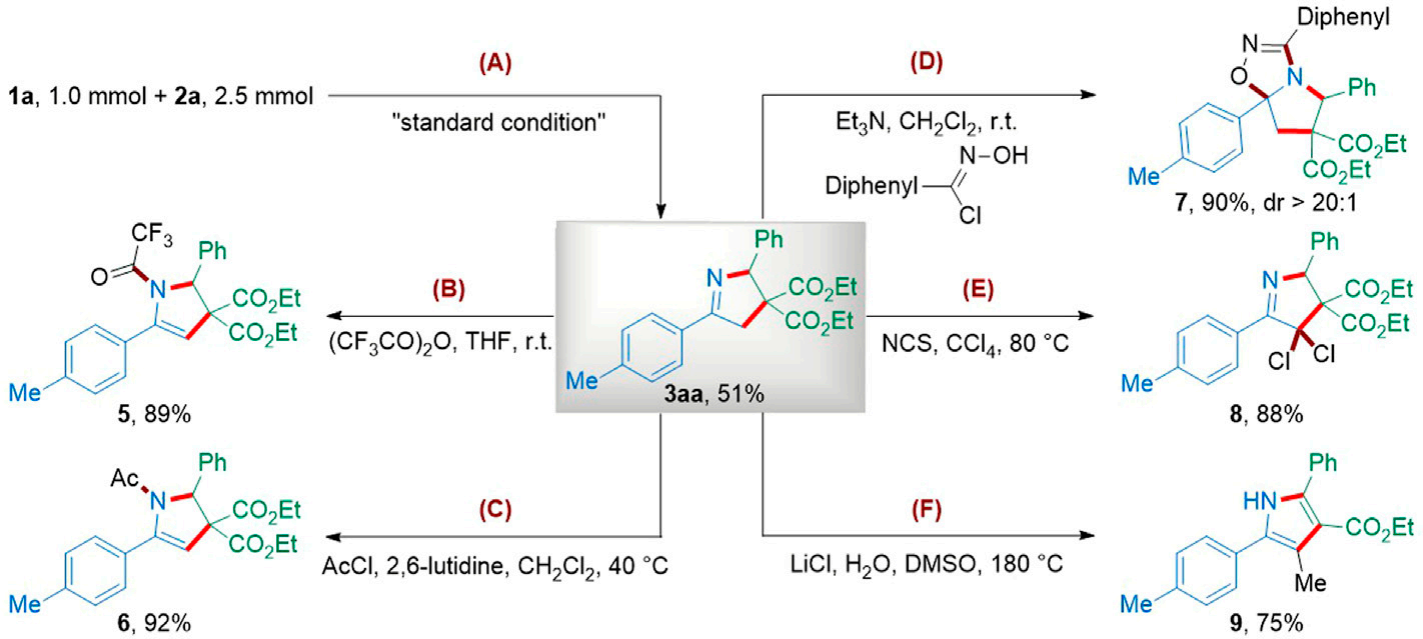
Representative derivatizations.

To illustrate the potential application of this protocol, we performed a scale-up reaction containing 1.0 mmol of **1a** under standard conditions to obtain the target product **3aa** with a synthetically useful yield (Scheme 4A). To further showcase the synthetic utility of this protocol, we next performed a set of facile derivatization applications using the formed 1-pyrroline **3aa**. In the presence of trifluoroacetic anhydride or acetyl chloride, **3aa** could be readily transformed into the acylated 2-pyrroline derivatives (**5** and **6**) with 89% and 92% yields, respectively (Schemes 4B,C). Additionally, [3 + 2] cycloaddition of **3aa** with *N*-hydroxybenzimidyl chloride successfully obtained biologically relevant pyrroline-fused 1,2,4-oxadiazoline **7** in an excellent yield of 90% (Scheme 4D). Upon treatment of **3aa** with *N*-chlorosuccinimide (NCS) at 80 °C, the *α*,*α*-dichlorinated 1-pyrroline derivative **8** was isolated in very good yield (Scheme 4E). Interestingly, treatment of **3aa** with LiCl in DMSO and trace H_2_O at 180°C resulted in the mono-decarboxylation, aromatization, and unclarified methylation process to afford tetra-substituted pyrrole **9** in moderate yield (Scheme 4F).

**SCHEME 5:**
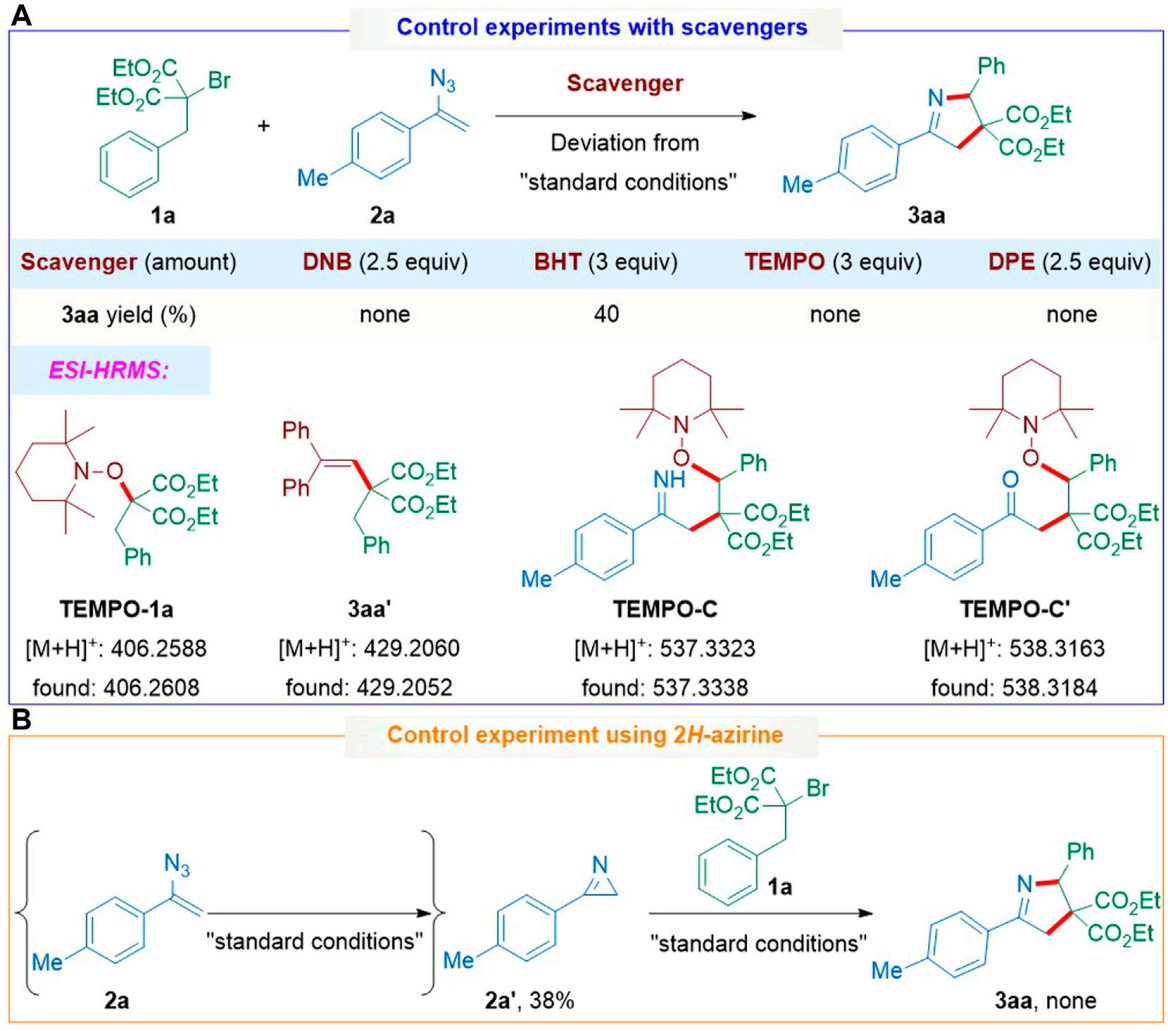
Preliminary mechanistic studies.

Subsequently, some control experiments were conducted to elucidate the reaction mechanism as shown in Scheme 5. When the electron-transfer scavenger p-dinitrobenzene (DNB) or radical scavenger including 2,6-di-*tert*-butyl-4-methylphenol (BHT), 2,2,6,6-tetramethyl-piperidinyloxyl (TEMPO), and 1,1-diphenylethylene (DPE) was added into the reaction system, the model reaction was suppressed to varying degrees (Scheme 5A). Notably, the existence of both **1a**-derived adduct **TEMPO-1a** detected by LC-HRMS and **3aa′** isolated by the chromatographic purification indicated that the corresponding alkyl radical might be generated from **1a**. Furthermore, a trace amount of other TEMPO-trapped adducts (**TEMPO-C** and **TEMPO-C′**) were also detected by LC-HRMS analysis, suggesting that the carbon-radical intermediates generated through the radical addition followed by iminyl radical-triggered 1,5-HAT might be involved in such transformation. Additionally, no desired product **3aa** was observed when using 3-phenyl-2*H*-azidocyclopropane **2a′** generated under standard conditions instead of vinyl azide **2a**, which seems to exclude the participation of 2*H*-aziridine intermediate in this transformation (Scheme 5B).

**SCHEME 6:**
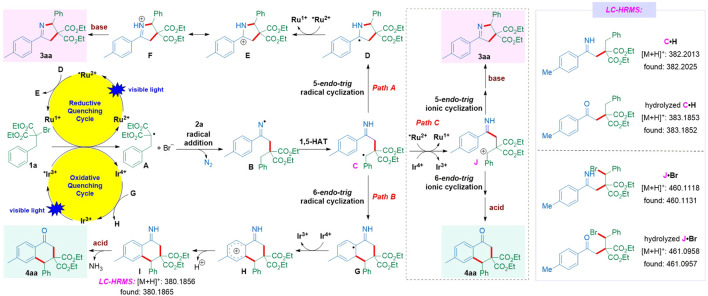
Plausible reaction mechanism.

Based on the above-mentioned results and literature survey, the reaction mechanism of [3 + 2] cyclization is depicted in Scheme 6. MacMillan group (Nicewicz and MacMillan, 2008) and Stephenson group (Wallentin et al., 2012; Swift et al., 2016) disclosed that, under the irradiation of visible light, the weakly reducing excited-state *Ru^2+^ [*E*_1/2_^red^ (Ru^3+^/*Ru^2+^) = −0.81 V vs. SCE] (Teegardin et al., 2016) can not be oxidatively quenched by the electron-deficient diethyl bromomalonate [*E*_1/2_^red^ = −1.0 V vs. SCE (Wang et al., 2016); while our model substrate diethyl 2-benzyl-2-bromomalonate **1a** (*E*_1/2_^red^ = −1.27 V vs. SCE) see the Supplementary Material]. In contrast, single electron transfer from the photogenerated electron-rich Ru^1+^ (−1.33 V vs. SCE) to **1a** could deliver an electrophilic alkyl radical **A**, although that the excited-state *Ru^2+^ [*E*_1/2_^red^ (*Ru^2+^/Ru^1+^) = +0.77 V vs. SCE] might be reductively quenched by certain unclarified reductant to initiate the first photocatalytic cycle and provide Ru^1+^ has not been clarified in detail yet. Subsequently, the addition of radical **A** onto vinyl azide **2a** leads to the release of an N_2_ molecule and the formation of an iminyl radical **B**, which undergoes 1,5-H atom transfer (1,5-HAT) to produce a more stabilized benzyl radical **C**. In path A, radical intermediate **C** proceeds via the 5-*endo*-*trig* radical cyclization with an imine moiety to give the electron-rich α-aminoalkyl radical **D** (–0.92 to –1.12 V vs. SCE) (Wayner et al., 1986; Du et al., 2020; Liang et al., 2020; Wu et al., 2020), which is readily oxidized by the excited-state *Ru^2+^ to access iminium cation **E** and finally yield the target 1-pyrroline **3aa** with the aid of a base. For the [4 + 2] cyclization, the strongly reducing excited-state *Ir^3+^ [*E*_1/2_^red^ (Ir^4+^/*Ir^3+^) = −1.73 V vs. SCE] (Teegardin et al., 2016) could be oxidatively quenched by diethyl 2-benzyl-2-bromomalonate **1a** to give radical intermediate **C** through the same radical addition/1,5-HAT sequence. As depicted in path B, intermediate **C** undergoes the 6-*endo*-*trig* radical cyclization onto the phenyl ring of vinyl azide to form **G**, which is further oxidized by the oxidized ground-state Ir^4+^ (+0.77 V vs. SCE) and then undergoes the deprotonation/hydrolysis process to release 1-tetralone **4aa** with the assistance of AcOH. In addition, another alternative path C, in which benzyl radical **C** could also undergo the single electron transfer with *Ru^2+^ or Ir^4+^ having a higher oxidation potential followed by 5- or 6-*endo*-*trig* ionic cyclization and further transformation to access **3aa** or **4aa**, could not be completely ruled out. In the [4 + 2] cyclization reaction, the additive acid might suppress the oxidation of α-aminoalkyl radical **D** in path A or 5-*endo*-trig ionic cyclization in path C, which resulted in the low efficiency of the [3 + 2] cyclization reaction. Notably, compound **C⋅H**, hydrolyzed **C⋅H**, compound **J⋅Br**, and hydrolyzed **J⋅Br** detected through LC-HRMS analysis of the ongoing reaction mixture validate the existence of radical **C** and cation **J**, providing further evidence for our proposed mechanism (for details see the Supplementary Material).

## Conclusion

In conclusion, we have developed a controllable photoredox-neutral [3 + 2] and [4 + 2] cyclization of alkyl bromides and vinyl azides by simply manipulating the photocatalysts and additives, providing chemodivergent access to highly substituted 1-pyrrolines and 1-tetralones. This protocol relies on intermolecular radical addition triggered 1,5-hydrogen atom transfer/switchable C(sp^3^)–H bond functionalization tandem sequences. In addition, the mild reaction conditions, easy-to-handle feedstocks, good tolerance of functional groups, easy scalability, facile derivatization of products, and late-stage functionalization of bioactive molecule provide great potential for application in synthetic chemistry and pharmaceutical chemistry. Further mechanistic research and application of this protocol are currently undergoing in our laboratory.

## Data Availability

The datasets presented in this study can be found in online repositories. The names of the repository/repositories and accession number(s) can be found in the article/Supplementary Material.
