# Supplementary material for: Chemodivergent photocatalytic access to 1-pyrrolines and 1-tetralones involving switchable C(sp3)–H functionalization
Source: Front Chem. 2022 Oct 25;10:1058596. doi: 10.3389/fchem.2022.1058596 (PMC9641198; doi:10.3389/fchem.2022.1058596)
Supplement: Supplementary file 2 [file DataSheet3.DOCX]

Supplementary Material

Contents

[1. General remarks S1](#_Toc112269427)

[2. The synthesis of the starting materials S2](#_Toc112269428)

[3. General procedure and product characterizations S5](#_Toc112269429)

[4. Synthetic applications of the reaction S34](#_Toc112269430)

[6. X-ray crystal data S49](#_Toc112269431)

[7. Supplementary references S51](#_Toc112269432)

[8. Copies of NMR spectra for products S52](#_Toc112269433)

# 1. General remarks

All reagents and starting materials, unless otherwise noted, were purchased from Energy, J&K, TCI, Alfa, Sigma-Aldrich, Innochem, and Adamas-beta^®^ Chemical Company as reagent grade and used without further purification. Anhydrous solvents (including DMSO, MeCN, DME, MeOH, CH_2_Cl_2_, DMF, ClCH_2_CH_2_Cl, THF, Water < 0.005%) were purchased from J&K and Energy, and used as received. Unless otherwise indicated, all syntheses and manipulations were carried out under an argon atmosphere.

^1^H, ^13^C, and ^19^F NMR spectra were obtained with a Bruker AV II-400 spectrometer (^1^H: 400 MHz, ^13^C: 101 MHz, ^19^F: 376 MHz). The ^1^H and ^13^C chemical shifts were measured relative to tetramethylsilane as the internal reference. The following abbreviations were used to explain the multiplicities: s = singlet, d = doublet, t = triplet, q = quartet, m = multiplet. TLC was performed using commercially prepared silica gel plates (GF254) and visualized by UV light 254 nm or iodine in silica gel. Flash column chromatography was performed on silica gel (100-200 mesh). Melting points were measured using a Hanon MP470 apparatus and were uncorrected. Cyclic voltammetry tests were carried out with a CHI700E electrochemical workstation. Mass analysis data were acquired on a Waters UPLC (H-Class) – QTOF(G2-XS) and Agilent UPLC (1290) – QTOF (6550).

# 2. The synthesis of the starting materials

**2.1 The preparation of** **alkyl bromides**

Alkyl bromides were synthesized according to the literature procedures.(Arceo et al., 2014) An oven-dried 100 mL round-bottom flask equipped with a stirring bar was charged with K_2_CO_3_ (11 mmol, 1.1 equiv.). The flask was connected to a vacuum line where it was evacuated and back-filled with Ar for three times. Next, diethyl 2-bromomalonate (10 mmol, 1.0 equiv.), (bromomethyl)benzene (12 mmol, 1.2 equiv.) and dry DMF (N,N-dimethylformamide) (20 mL) were added under Ar flow. Then stirred at room temperature for 9 h. After completion, the mixture was poured into a separatory funnel containing EtOAc and H_2_O. The layers were separated and the aqueous layer was extracted with EtOAc. The organic layer was washed with saturated brine and dried over Na_2_SO_4_. The solvent was removed under reduced pressure, and the resulting residue was purified by column chromatography on silica gel to afford the desired product (**1a-1t**).

Alkyl bromides were synthesized according to the literature procedures.(Jiao et al., 2021) An oven-dried 100 mL round-bottom flask equipped with a stirring bar was charged with Mg(ClO_4_)_2_ (3.0 mmol, 0.3 equiv.). The flask was connected to a vacuum line where it was evacuated and back-filled with Ar for three times. Next, diethyl 2-alkylmalonate (10mmol, 1.0 equiv.) and dry EtOAc (30 mL) were added under Ar flow. Then stirred at room temperature for 0.5 h. Then, NBS (11 mmol, 1.1 equiv.) was added to the above mixture. After completion, the mixture was poured into a separatory funnel containing EtOAc and H_2_O. The layers were separated and the aqueous layer was extracted with EtOAc. The organic layer was washed with saturated brine and dried over Na_2_SO_4_. The solvent was removed under reduced pressure, and the resulting residue was purified by column chromatography on silica gel to afford the desired product (**1u** and **1v**).

**2.2 The preparation of vinyl azides**

Vinyl azides were synthesized according to the literature procedures.(Liu et al., 2014) An oven-dried 100 mL round-bottom flask equipped with a stirring bar was charged with AgCO_3_ (0.5 mmol, 0.1 equiv.). The flask was connected to a vacuum line where it was evacuated and back-filled with Ar for three times. Next, alkyne (5 mmol, 1.0 equiv.), TMS-N_3_ (10 mmol, 2.0 equiv.), H_2_O (10 mmol, 2.0 equiv.), and dry DMSO (20 mL) were added under Ar flow. Then stirred at 80℃ for 9 h. After completion, the mixture was poured into a separatory funnel containing EtOAc and H_2_O. The layers were separated and the aqueous layer was extracted with EtOAc. The organic layer was washed with saturated brine and dried over Na_2_SO_4_. The solvent was removed under reduced pressure, and the resulting residue was purified by column chromatography on silica gel to afford the desired product.

# 3. General procedure and product characterizations

**3.1 Photocatalytic [3+2] cyclization to access 1-pyrrolines**

An oven-dried Schlenk tube (10 mL) equipped with a stirring bar was charged with alkyl bromide **1** (0.2 mmol), K_2_CO_3_ (0.3 mmol, 1.5 equiv.), and Ru(bpy)_3_(PF_6_)_2_ (2 mol%). The tube was connected to a vacuum line where it was evacuated and back-filled with Ar for three times. Next, vinyl azide **2** (0.5 mmol, 2.5 equiv.) and dry CH_2_Cl_2_ (2 mL) were added under Ar flow. The tube was placed approximately 2 cm from 30 W blue LEDs, and then stirred at room temperature for 9 h. After completion, the reaction was quenched with H_2_O (6 mL) and extracted with EtOAc. The organic layer was washed with saturated brine and dried over Na_2_SO_4_. The solvent was removed under reduced pressure, and the resulting residue was purified by column chromatography on silica gel to afford the desired product.

**diethyl 2-phenyl-5-(*p*-tolyl)-2,4-dihydro-3*H*-pyrrole-3,3-dicarboxylate (3aa)**

59.9 mg, 79% (eluent: PE/EA 20/1);

Appearance: yellow oil;

**^1^H NMR (400 MHz, CDCl_3_)** δ 7.84 (d, *J* = 7.8 Hz, 2H), 7.26 – 7.24 (m, 7H), 6.29 (s, 1H), 4.34 – 4.20 (m, 2H), 4.14 – 4.09 (m, 1H), 3.71 – 3.66 (m, 1H), 3.45 – 3.39 (m, 2H), 2.40 (s, 3H), 1.29 (t, *J* = 7.1 Hz, 3H), 0.82 (t, *J* = 7.1 Hz, 3H).

**^13^C NMR (101 MHz, CDCl_3_)** δ 171.0, 170.5, 169.0, 141.5, 138.1, 130.6, 129.3, 128.2, 128.0, 127.9, 127.8, 80.6, 65.0, 62.0, 61.6, 44.1, 21.6, 14.1, 13.4.

**HRMS (ESI)** calcd for C_23_H_25_NO_4_ [M+H]^+^: 380.1856, found: 380.1862.

**diethyl 2-(4-methoxyphenyl)-5-(*p*-tolyl)-2,4-dihydro-3*H*-pyrrole-3,3-dicarboxylate (3ba)**

35.2 mg, 43 % (eluent: PE/EA 20/1);

Appearance: yellow solid.;

Melting point**:** 133.8 – 134.7 °C;

**^1^H NMR (400 MHz, CDCl_3_)** δ 7.76 (d, *J* = 7.9 Hz, 2H), 7.18 (d, *J* = 6.7 Hz, 2H), 7.09 (d, *J* = 8.6 Hz, 2H), 6.73 (d, *J* = 8.7 Hz, 2H),

6.18 (s, 1H), 4.27 – 4.13 (m, 2H), 4.05 – 4.00 (m, 1H), 3.69 (s, 3H), 3.68 – 3.65 (m, 1H), 3.44 – 3.33 (m, 2H), 2.34 (s, 3H), 1.22 (t, *J* = 7.1 Hz, 3H), 0.81 (t, *J* = 7.1 Hz, 3H).

**^13^C NMR (101 MHz, CDCl_3_)** δ 171.02, 170.26, 168.99, 158.76, 140.84, 130.56, 130.09, 129.33, 129.31, 128.00, 113.33, 80.06, 64.86, 62.02, 61.58, 55.28, 43.92, 21.56, 14.06, 13.55.

**HRMS (ESI)** calcd for C_24_H_27_NO_5_ [M+H]^+^: 410.1962, found: 410.1964.

**diethyl 2,5-di-*p*-tolyl-2,4-dihydro-3*H*-pyrrole-3,3-dicarboxylate (3ca)**

43.2 mg, 55% (eluent: PE/EA 20/1);

Appearance: yellow oil;

**^1^H NMR (400 MHz, CDCl_3_)** δ 7.83 (d, *J* = 7.8 Hz, 2H), 7.25 (d, *J* = 7.7 Hz, 2H), 7.12 (d, *J* = 7.6 Hz, 2H), 7.07 (d, *J* = 7.9 Hz, 2H), 6.26 (s, 1H), 4.35 – 4.18 (m, 2H), 4.09 (d, *J* = 17.8 Hz, 1H), 3.75 – 3.67 (m, 1H), 3.50 – 3.40 (m, 2H), 2.41 (s, 3H), 2.29 (s, 3H), 1.29 (t, *J* = 7.3 Hz, 3H), 0.85 (t, *J* = 7.3 Hz, 3H).

**^13^C NMR (101 MHz, CDCl_3_)** δ 171.04, 170.24, 168.97, 141.42, 137.38, 134.95, 130.71, 129.27, 128.58, 128.05, 127.96, 80.43, 64.97, 61.98, 61.52, 43.95, 21.54, 21.15, 14.05, 13.45.

**HRMS (ESI)** calcd for C_24_H_27_NO_4_ [M+H]^+^: 394.2013, found: 394.2020.

**diethyl 2-(4-(tert-butyl)phenyl)-5-(*p*-tolyl)-2,4-dihydro-3*H*-pyrrole-3,3-dicarboxylate (3da)**

52.2 mg, 60% (eluent: PE/EA 20/1);

Appearance: yellow oil;

**^1^H NMR (400 MHz, CDCl_3_)** δ 7.83 (d, *J* = 8.1 Hz, 2H), 7.29-7.24 (m, 4H), 7.16 (d, *J* = 8.2 Hz, 2H), 6.25 (s, 1H), 4.33 – 4.18 (m, 2H), 4.12 (d, *J* = 17.8 Hz, 1H), 3.69 – 3.61

(m, 1H), 3.48 – 3.39 (m, 2H), 2.41 (s, 3H), 1.30 – 1.28 (m, 3H), 1.26 (s, 9H), 0.74 (t, *J* = 7.2 Hz, 3H).

**^13^C NMR (101 MHz, CDCl_3_)** δ 171.1, 170.1, 169.1, 150.7, 141.4, 135.1, 130.7, 129.3, 128.0, 127.9, 124.8, 80.5, 64.9, 62.0, 61.6, 44.1, 34.5, 31.3, 21.6, 14.1, 13.3.

**HRMS (ESI)** calcd for C_27_H_33_NO_4_ [M+H]^+^: 436.2482, found: 436.2492.

**diethyl 2-(4-fluorophenyl)-5-(*p*-tolyl)-2,4-dihydro-3*H*-pyrrole-3,3-dicarboxylate (3ea)**

38.1 mg, 48% (eluent: PE/EA 20/1);

Appearance: yellow oil;

**^1^H NMR (400 MHz, CDCl_3_)** δ 7.85 (d, *J* = 7.7 Hz, 2H), 7.29 – 7.27 (m, 4H), 7.01 – 6.98 (m, 2H), 6.28 (s, 1H), 4.37 – 4.22 (m, 2H), 4.14 – 4.10 (m, 1H), 3.80 – 3.71 (m, 1H), 3.53 – 3.42 (m, 2H), 2.44 (s, 3H), 1.32 (t, *J* = 7.2 Hz, 3H), 0.89 (t, *J* = 7.2 Hz, 3H).

**^13^C NMR (101 MHz, CDCl_3_)** δ 170.9, 170.7, 168.9, 162.5 (d, *J* = 245.9 Hz), 141.7, 134.1 (d, *J* = 3.2 Hz), 130.5, 129.9 (d, *J* = 8.1 Hz), 129.3, 127.9, 114.8 (d, *J* = 21.3 Hz), 79.8, 64.9, 62.1, 61.6, 44.2, 21.6, 14.1, 13.5.

**^19^F NMR (376 MHz, CDCl_3_)** δ -114.76.

**HRMS (ESI)** calcd for C_23_H_24_FNO_4_ [M+H]^+^: 398.1762, found: 398.1769.

**diethyl 2-(4-chlorophenyl)-5-(*p*-tolyl)-2,4-dihydro-3*H*-pyrrole-3,3-dicarboxylate (3fa)**

66.9 mg, 81% (eluent: PE/EA 20/1);

Appearance: yellow oil;

**^1^H NMR (400 MHz, CDCl_3_)** δ 7.75 (d, *J* = 8.2 Hz, 2H), 7.21 – 7.10 (m, 6H), 6.16 (s, 1H), 4.28 – 4.19 (m, 2H), 4.04 – 3.99 (m, 1H), 3.68 – 3.63 (m, 1H), 3.45 – 3.41 (m, 1H), 3.38 – 3.33 (m, 1H), 2.33 (s, 3H), 1.23 (t, *J* = 7.1 Hz, 3H), 0.80 (t, *J* = 7.1 Hz, 3H).

**^13^C NMR (101 MHz, CDCl_3_)** δ 171.2, 170.7, 168.8, 141.8, 140.5, 133.8, 130.4, 129.4, 129.2, 128.4, 128.0, 127.9, 126.3, 79.9, 64.9, 62.2, 61.8, 44.3, 21.6, 14.1, 13.4.

**HRMS (ESI)** calcd for C_23_H_24_ClNO_4_ [M+H]^+^: 414.1467, found: 414.1473.

**diethyl 2-(4-bromophenyl)-5-(*p*-tolyl)-2,4-dihydro-3*H*-pyrrole-3,3-dicarboxylate (3ga)**

71.3 mg, 78% (eluent: PE/EA 20/1);

Appearance: yellow solid.

Melting point: 105.8 – 107.8 °C.

**^1^H NMR (400 MHz, CDCl_3_)** δ 7.82 (d, *J* = 7.7 Hz, 2H), 7.41 (d, *J* = 7.6 Hz, 2H), 7.25 (d, *J* = 7.4 Hz, 2H), 7.17 (d, *J* = 8.0 Hz, 2H),

6.22 (s, 1H), 4.38 – 4.18 (m, 2H), 4.08 (d, *J* = 17.8 Hz, 1H), 3.79 – 3.68 (m, 1H), 3.54 – 3.38 (m, 2H), 2.41 (s, 3H), 1.29 (t, *J* = 7.1 Hz, 3H), 0.87 (t, *J* = 6.9 Hz, 3H).

**^13^C NMR (101 MHz, CDCl_3_)** δ 171.0, 170.8, 168.8, 141.7, 137.5, 131.0, 130.4, 129.9, 129.4, 128.0, 121.8, 79.8, 64.9, 62.2, 61.7, 44.3, 21.6, 14.1, 13.5.

**HRMS (ESI)** calcd for C_23_H_24_BrNO_4_ [M+H]^+^: 458.0961, found: 458.0966.

**diethyl 2-(4-cyanophenyl)-5-(*p*-tolyl)-2,4-dihydro-3*H*-pyrrole-3,3-dicarboxylate (3ha)**

33.9 mg, 42% (eluent: PE/EA 20/1);

Appearance: yellow solid;

Melting point**:** 110.9 – 112.6 °C;

**^1^H NMR (400 MHz, CDCl_3_)** δ 7.74 (d, *J* = 8.2 Hz, 2H), 7.52 (d, *J* = 8.4 Hz, 2H), 7.39 (d, *J* = 8.4 Hz, 2H), 7.22 – 7.17 (m, 2H), 6.19 (s, 1H), 4.38 – 4.13 (m, 2H), 4.07 – 3.96 (m, 1H), 3.69 – 3.56 (m, 1H), 3.42 – 3.32 (m, 2H), 2.35 (s, 3H), 1.23 (t, *J* = 7.1 Hz, 3H), 0.76 (t, *J* = 7.2 Hz, 3H).

**^13^C NMR (101 MHz, CDCl_3_)** δ 171.2, 170.7, 168.8, 141.8, 140.8, 131.3, 130.8, 129.5, 129.4, 128.0, 126.8, 122.0, 79.9, 65.0, 62.2, 61.8, 44.3, 21.6, 14.0, 13.5.

**HRMS (ESI)** calcd for C_24_H_24_N_2_O_4_ [M+H]^+^: 405.1809, found: 405.1809.

**diethyl 2-(4-acetylphenyl)-5-(*p*-tolyl)-2,4-dihydro-3*H*-pyrrole-3,3-dicarboxylate (3ia)**

40.4 mg, 48% (eluent: PE/EA 20/1);

Appearance: yellow oil;

**^1^H NMR (400 MHz, CDCl_3_)** δ 7.89 (d, *J* = 7.7 Hz, 2H), 7.84 (d, *J* = 7.9 Hz, 2H), 7.40 (d, *J* = 7.6 Hz, 2H), 7.27 (d, *J* = 7.1 Hz, 2H), 6.31 (s, 1H), 4.40 – 4.20 (m, 2H), 4.11 (d, *J* = 17.8 Hz, 1H), 3.75 – 3.62 (m, 1H), 3.52 – 3.36 (m, 2H), 2.59 (s, 3H), 2.42 (s, 3H), 1.31 (t, *J* = 7.1 Hz, 3H), 0.82 (t, *J* = 7.1 Hz, 3H).

**^13^C NMR (101 MHz, CDCl_3_)** δ 197.9, 171.3, 170.7, 168.8, 143.9, 141.8, 136.5, 130.4, 129.4, 128.5, 128.0, 80.1, 65.0, 62.2, 61.7, 44.4, 26.7, 21.6, 14.1, 13.5.

**HRMS (ESI)** calcd for C_25_H_27_NO_5_ [M+H]^+^: 422.1962, found: 422.1968.

**diethyl 2-(3-fluorophenyl)-5-(*p*-tolyl)-2,4-dihydro-3*H*-pyrrole-3,3-dicarboxylate (3ja)**

57.2 mg, 72% (eluent: PE/EA 20/1);

Appearance: yellow oil;

**^1^H NMR (400 MHz, CDCl_3_)** δ 7.83 (d, *J* = 6.8 Hz, 2H), 7.30 – 7.21 (m, 3H), 7.09 (d, *J* = 7.7 Hz, 1H), 7.01 (d, *J* = 7.8 Hz, 1H), 6.96 – 6.92 (m, 1H)., 6.25 (s, 1H), 4.39 – 4.20 (m, 2H), 4.08 (d, *J* = 17.7 Hz, 1H), 3.80 – 3.68 (m, 1H), 3.56 – 3.39 (m, 2H), 2.41 (s, 3H), 1.30 (t, *J* = 7.2 Hz, 3H), 0.86 (t, *J* = 7.2 Hz, 3H).

**^13^C NMR (101 MHz, CDCl_3_)** δ 171.1, 170.7, 168.8, 162.6 (d, *J* = 245.4 Hz), 141.7, 141.0 (d, *J* = 7.1 Hz), 130.5, 129.37 (d, *J* = 7.4 Hz), 129.3, 128.0, 123.9 (d, *J* = 2.9 Hz), 115.2 (d, *J* = 22.0 Hz), 114.6 (d, *J* = 21.2 Hz), 79.9, 64.9, 62.1, 61.7, 44.3, 21.5, 14.0, 13.4.

**^19^F NMR (376 MHz, CDCl_3_)** δ -113.76.

**HRMS (ESI)** calcd for C_23_H_24_FNO_4_ [M+H]^+^: 398.1762, found: 398.1767.

**diethyl 2-(3-chlorophenyl)-5-(*p*-tolyl)-2,4-dihydro-3*H*-pyrrole-3,3-dicarboxylate (3ka)**

57.0 mg, 69% (eluent: PE/EA 20/1);

Appearance: yellow oil;

**^1^H NMR (400 MHz, CDCl_3_)** δ 7.83 (d, *J* = 7.8 Hz, 2H), 7.30 – 7.18 (m, 6H), 6.23 (s, 1H), 4.35 – 4.22 (m, 2H), 4.09 (d, *J* = 17.9 Hz, 1H), 3.79 – 3.68 (m, 1H), 3.56 – 3.47 (m, 1H), 3.43 (d, *J* = 17.9 Hz, 1H), 2.41 (s, 3H), 1.30 (t, *J* = 6.9 Hz, 3H), 0.87 (t, *J* = 6.9 Hz, 3H).

**^13^C NMR (101 MHz, CDCl_3_)** δ 171.2, 170.7, 168.8, 141.7, 140.5, 133.8, 130.4, 129.3, 129.2, 128.4, 128.0, 127.9, 126.3, 79.9, 64.9, 62.2, 61.7, 44.3, 21.6, 14.0, 13.4.

**HRMS (ESI)** calcd for C_23_H_24_ClNO_4_ [M+H]^+^: 414.1467, found: 414.1469.

**diethyl 2-(3-bromophenyl)-5-(*p*-tolyl)-2,4-dihydro-3*H*-pyrrole-3,3-dicarboxylate (3la)**

51.2 mg, 56% (eluent: PE/EA 20/1);

Appearance: yellow oil;

**^1^H NMR (400 MHz, CDCl_3_)** δ 7.75 (d, *J* = 7.6 Hz, 2H), 7.38 (s, 1H), 7.31 (d, *J* = 7.8 Hz, 1H), 7.20 – 7.08 (m, 4H), 6.15 (s, 1H), 4.28 – 4.15 (m, 2H), 4.01 (d, *J* = 17.9 Hz, 1H), 3.70 – 3.62 (m, 1H), 3.48 – 3.41 (m, 1H), 3.36 (d, *J* = 17.8 Hz, 1H), 2.34 (s, 3H), 1.23 (t, *J* = 7.3 Hz, 3H), 0.82 (t, *J* = 7.3 Hz, 3H).

**^13^C NMR (101 MHz, CDCl_3_)** δ 171.2, 170.7, 168.8, 141.8, 140.8, 131.3, 130.8, 130.4, 129.5, 129.4, 128.0, 126.8, 122.0, 79.9, 65.0, 62.2, 61.8, 44.3, 21.6, 14.0, 13.5.

**HRMS (ESI)** calcd for C_23_H_24_BrNO_4_ [M+H]^+^: 458.0961, found: 458.0965.

**diethyl 2-(3-methoxyphenyl)-5-(*p*-tolyl)-2,4-dihydro-3*H*-pyrrole-3,3-dicarboxylate (3ma)**

37.6 mg,46% (eluent: PE/EA 20/1);

Appearance: yellow oil;

**^1^H NMR (400 MHz, CDCl_3_)** δ 7.83 (d, *J* = 7.8 Hz, 2H), 7.26 (d, *J* = 7.5 Hz, 2H), 7.20-7.16 (m, 1H), 6.85 (d, *J* = 7.7 Hz, 1H), 6.80 – 6.78 (m, 2H), 6.25 (s, 1H), 4.38 – 4.18 (m, 2H), 4.15 – 4.06 (m, 1H), 3.76 (s, 3H), 3.74 – 3.67 (m, 1H), 3.53 – 3.39 (m, 2H), 2.41 (s, 3H), 1.29 (t, *J* = 7.2 Hz, 3H), 0.85 (t, *J* = 7.3 Hz, 3H).

**^13^C NMR (101 MHz, CDCl_3_)** δ 171.0, 170.5, 168.9, 159.3, 141.5, 139.7, 130.6, 129.3, 128.9, 128.0, 120.7, 113.7, 113.5, 80.5, 64.9, 62.1, 61.6, 55.3, 44.1, 21.6, 14.1, 13.5.

**HRMS (ESI)** calcd for C_24_H_27_NO_5_ [M+H]^+^: 410.1962, found: 410.1968.

**diethyl 2-(*o*-tolyl)-5-(*p*-tolyl)-2,4-dihydro-3*H*-pyrrole-3,3-dicarboxylate (3na)**

55.0 mg, 70% (eluent: PE/EA 20/1);

Appearance: yellow oil;

**^1^H NMR (400 MHz, CDCl_3_)** δ 7.83 (d, *J* = 7.8 Hz, 2H), 7.25 (d, *J* = 8.3 Hz, 2H), 7.11 (d, *J* = 7.7 Hz, 2H), 7.06–7.02 (m, 1H), 6.78 (d, *J* = 7.7 Hz, 1H), 6.69 (s, 1H), 4.31 – 4.17 (m, 3H), 3.78 – 3.66 (m, 1H), 3.49 – 3.45 (m, 1H), 3.41 – 3.30 (m, 1H), 2.53 (s, 3H), 2.41 (s, 3H), 1.28 (t, *J* = 7.2 Hz, 3H), 0.85 (t, *J* = 7.2 Hz, 3H).

**^13^C NMR (101 MHz, CDCl_3_)** δ 171.2, 169.9, 168.8, 141.5, 137.0, 136.3, 130.6, 130.1, 129.3, 128.0, 127.7, 127.6, 125.7, 77.1, 64.4, 62.1, 61.6, 44.4, 21.5, 20.1, 14.0, 13.4.

**HRMS (ESI)** calcd for C_24_H_27_NO_4_ [M+H]^+^: 394.2013, found: 394.2015.

**diethyl 2-(2-fluorophenyl)-5-(*p*-tolyl)-2,4-dihydro-3*H*-pyrrole-3,3-dicarboxylate (3oa)**

36.5 mg, 46% (eluent: PE/EA 20/1);

Appearance: yellow oil;

**^1^H NMR (400 MHz, CDCl_3_)** δ 7.82 (d, *J* = 7.9 Hz, 2H), 7.32 – 7.18 (m, 4H), 7.06 – 6.94 (m, 2H), 6.68 (s, 1H), 4.40 – 4.27 (m, 1H), 4.27 – 4.14 (m, 2H), 3.85 – 3.71 (m, 1H), 3.55 – 3.44 (m, 2H), 2.40 (s, 3H), 1.28 (t, *J* = 7.2 Hz, 3H), 0.89 (t, *J* = 7.2 Hz, 3H).

**^13^C NMR (101 MHz, CDCl_3_)** δ 171.33, 170.56, 168.69, 160.65 (d, *J* = 249.1 Hz), 141.95, 130.14, 129.72 (d, *J* = 3.8 Hz), 129.71 (d, *J* = 7.9 Hz), 129.40, 128.15, 125.29 (d, *J* = 13.4 Hz), 123.88 (d, *J* = 3.6 Hz), 115.25 (d, *J* = 22.1 Hz), 74.11, 64.14, 62.22, 61.76, 44.36, 21.59, 14.04, 13.41.

**^19^F NMR (376 MHz, CDCl_3_)** δ -115.15.

**HRMS (ESI)** calcd for C_23_H_24_FNO_4_ [M+H]^+^: 398.1762, found: 398.1763.

**diethyl 2-(2-chlorophenyl)-5-(*p*-tolyl)-2,4-dihydro-3*H*-pyrrole-3,3-dicarboxylate (3pa)**

50.4 mg, 61% (eluent: PE/EA 20/1);

Appearance: yellow oil;

**^1^H NMR (400 MHz, CDCl_3_)** δ 7.82 (d, *J* = 8.1 Hz, 2H), 7.36 (d, *J* = 7.6 Hz, 1H), 7.25 (d, *J* = 6.2 Hz, 2H), 7.20 – 7.08 (m, 2H), 6.95 (s, 1H), 6.88 (d, *J* = 7.5 Hz, 1H), 4.38 – 4.17 (m, 2H), 4.38 – 4.17 (m, 1H), 3.82 – 3.69 (m, 1H), 3.52 – 3.38 (m, 2H), 2.40 (s, 3H), 1.28 (t, *J* = 7.1 Hz, 3H), 0.91 (t, *J* = 7.2 Hz, 3H).

**^13^C NMR (101 MHz, CDCl_3_)** δ 171.2, 170.7, 168.8, 141.7, 140.5, 133.8, 130.4, 129.4, 129.3, 128.4, 128.0, 127.9, 126.3, 79.9, 64.9, 62.2, 61.8, 44.3, 21.6, 14.1, 13.4.

**HRMS (ESI)** calcd for C_23_H_24_ClNO_4_ [M+H]^+^: 414.1467, found: 414.1471

**diethyl 2-(3,5-dimethylphenyl)-5-(*p*-tolyl)-2,4-dihydro-3*H*-pyrrole-3,3-dicarboxylate (3qa)**

42.3 mg, 52% (eluent: PE/EA 20/1);

Appearance: yellow oil;

**^1^H NMR (400 MHz, CDCl_3_)** δ 7.85 (d, *J* = 8.2 Hz, 2H), 7.26 (d, *J* = 8.0 Hz, 2H), 6.85 (s, 1H), 6.82 (s, 2H), 6.22 (s, 1H), 4.34 – 4.20 (m, 2H), 4.14 – 4.08 (m, 1H), 3.75 – 3.66 (m, 1H), 3.51 – 3.45 (m, 1H), 3.41 (d, *J* = 17.7 Hz, 1H), 2.41 (s, 3H), 2.24 (s, 6H), 1.29 (t, *J* = 7.1 Hz, 3H), 0.84 (t, *J* = 7.1 Hz, 3H).

**^13^C NMR (101 MHz, CDCl_3_)** δ 171.1, 170.1, 168.9, 141.4, 137.8, 137.2, 130.7, 129.3, 129.3, 128.0, 126.0, 81.3, 64.9, 62.0, 61.5, 43.5, 21.5, 21.3, 14.1, 13.4.

**HRMS (ESI)** calcd for C_25_H_29_NO_4_ [M+H]^+^: 408.2169, found: 408.2174.

**diethyl 2-(1-(*tert*-butoxycarbonyl)-1*H*-indol-3-yl)-5-(*p*-tolyl)-2,4-dihydro-3*H*-pyrrole-3,3-dicarboxylate (3ra)**

67.3 mg, 65% (eluent: PE/EA 20/1);

Appearance: yellow oil;

**^1^H NMR (400 MHz, CDCl_3_)** δ 8.09 (d, *J* = 8.2 Hz, 1H), 7.84 (d, *J* = 8.2 Hz, 2H), 7.71 (d, *J* = 8.8 Hz, 1H), 7.33 (s, 1H), 7.29 – 7.24 (m, 3H), 7.23 – 7.18 (m, 1H), 6.62 (s, 1H), 4.38 – 4.24 (m, 2H), 4.23 – 4.17 (m, 1H), 3.64 – 3.60 (m, 1H), 3.54 – 3.47 (m, 1H), 3.25 – 3.17 (m, 1H), 2.42 (s, 3H), 1.62 (s, 9H), 1.31 (t, *J* = 7.1 Hz, 3H), 0.61 (t, *J* = 7.1 Hz, 3H).

**^13^C NMR (101 MHz, CDCl_3_)** δ 171.0, 170.4, 169.0, 149.6, 141.6, 130.6, 129.6, 129.3, 128.0, 124.6, 124.3, 122.4, 120.4, 118.3, 114.9, 83.7, 73.5, 64.2, 62.1, 61.5, 44.1, 28.2, 21.6, 14.1, 13.0.

**HRMS (ESI)** calcd for C_30_H_34_N_2_O_6_ [M+H]^+^: 519.2490, found: 519.2487.

**diethyl 2-(2-methylprop-1-en-1-yl)-5-(*p*-tolyl)-2,4-dihydro-3*H*-pyrrole-3,3-dicarboxylate (3sa)**

50.7 mg, 71% (eluent: PE/EA 20/1);

Appearance: yellow oil;

**^1^H NMR (400 MHz, CDCl_3_)** δ 7.73 (d, *J* = 8.1 Hz, 2H), 7.20 (d, *J* = 7.9 Hz, 2H), 5.94 (d, *J* = 10.4 Hz, 1H), 4.85 (d, *J* = 10.4 Hz, 1H), 4.26 – 4.07 (m, 4H), 4.02 – 3.96 (m, 1H), 3.42 – 3.37 (m, 1H), 2.37 (s, 3H), 1.86 (s, 3H), 1.70 (s, 3H), 1.25 (t, *J* = 7.1 Hz, 3H), 1.20 (t, *J* = 7.1 Hz, 3H).

**^13^C NMR (101 MHz, CDCl_3_)** δ 171.0, 169.4, 169.2, 141.2, 137.7, 130.8, 129.2, 127.8, 121.9, 74.9, 63.9, 61.9, 61.6, 43.3, 26.0, 21.5, 18.7, 14.1, 14.0.

**HRMS (ESI)** calcd for C_21_H_27_NO_4_ [M+H]^+^: 358.2013, found: 358.2014.

**diethyl 2-(prop-1-yn-1-yl)-5-(*p*-tolyl)-2,4-dihydro-3*H*-pyrrole-3,3-dicarboxylate (3ta)**

55.3 mg, 70% (eluent: PE/EA 20/1);

Appearance: yellow oil;

**^1^H NMR (400 MHz, CDCl_3_)** δ 7.75 (d, *J* = 7.9 Hz, 2H), 7.21 (d, *J* = 7.9 Hz, 2H), 5.80 (s, 1H), 4.32 – 4.14 (m, 4H), 4.06 – 3.98 (m, 1H), 3.42 – 3.32 (m, 1H), 2.38 (s, 3H), 1.84 – 1.74 (m, 3H), 1.35 – 1.27 (m, 6H).

**^13^C NMR (101 MHz, CDCl_3_)** δ 171.0, 170.1, 168.7, 141.6, 130.4, 129.2, 128.0, 82.9, 74.6, 68.4, 64.1, 62.2, 62.1, 43.1, 21.5, 14.1, 14.0, 3.8.

**HRMS (ESI)** calcd for C_20_H_23_NO_4_ [M+H]^+^: 342.1700, found: 342.1704.

**diethyl 5-(4-methoxyphenyl)-2-phenyl-2,4-dihydro-3*H*-pyrrole-3,3-dicarboxylate (3ab)**

55.3 mg, 70% (eluent: PE/EA 20/1);

Appearance: yellow oil;

**^1^H NMR (400 MHz, CDCl_3_)** δ 7.50 – 7.47 (m, 1H), 7.39 (d, *J* = 7.7 Hz, 1H), 7.31–7.27 (m, 1H), 7.21 – 7.16 (m, 5H), 7.00 – 6.93 (m, 1H), 6.23 (s, 1H), 4.32 – 4.13 (m, 2H), 4.10 – 4.02 (m, 1H), 3.78 (s, 3H), 3.66 – 3.59 (m, 1H), 3.40 – 3.36 (m, 1H), 3.36 – 3.32 (m, 1H), 1.22 (t, *J* = 7.1 Hz, 3H), 0.76 (t, *J* = 7.1 Hz, 3H).

**^13^C NMR (101 MHz, CDCl_3_)** δ 170.9, 170.5, 168.9, 159.7, 137.9, 134.6, 129.6, 128.2, 128.0, 127.9, 120.7, 117.9, 112.0, 80.7, 64.9, 62.1, 61.6, 55.5, 44.2, 14.1, 13.5.

**HRMS (ESI)** calcd for C_23_H_25_NO_5_ [M+H]^+^: 396.1805, found: 396.1812.

**diethyl 5-(4-ethylphenyl)-2-phenyl-2,4-dihydro-3*H*-pyrrole-3,3-dicarboxylate (3ac)**

51.9 mg, 66% (eluent: PE/EA 20/1);

Appearance: yellow oil;

**^1^H NMR (400 MHz, CDCl_3_)** δ 7.87 (d, *J* = 8.2 Hz, 2H), 7.31 – 7.23 (m, 7H), 6.29 (s, 1H), 4.35 – 4.20 (m, 2H), 4.16 – 4.07 (m, 1H), 3.73 – 3.64 (m, 1H), 3.47 – 3.39 (m, 2H), 2.70 (q, *J* = 7.6 Hz, 2H), 1.29 (t, *J* = 7.1 Hz, 3H), 1.26 (t, *J* = 7.6 Hz, 3H), 0.82 (t, *J* = 7.1 Hz, 3H).

**^13^C NMR (101 MHz, CDCl_3_)** δ 171.0, 170.5, 168.9, 147.8, 138.1, 130.9, 128.2, 128.1, 128.1, 127.9, 127.8, 80.6, 65.0, 62.0, 61.6, 44.1, 28.9, 15.4, 14.1, 13.4.

**HRMS (ESI)** calcd for C_24_H_27_NO_4_ [M+H]^+^: 394.2013, found: 394.2018.

**diethyl 2-phenyl-5-(4-propylphenyl)-2,4-dihydro-3*H*-pyrrole-3,3-dicarboxylate (3ad)**

61.1 mg, 75% (eluent: PE/EA 20/1);

Appearance: yellow oil;

**^1^H NMR (400 MHz, CDCl_3_)** δ 7.79 (d, *J* = 8.2 Hz, 2H), 7.22 – 7.15 (m, 7H), 6.22 (s, 1H), 4.29 – 4.12 (m, 2H), 4.09 – 3.99 (m, 1H), 3.66 – 3.57 (m, 1H), 3.41 – 3.29 (m, 2H), 2.56 (t, *J* = 7.6 Hz, 2H), 1.61 – 1.50 (m, 2H), 1.21 (t, *J* = 7.1 Hz, 3H), 0.88 (t, *J* = 7.3 Hz, 3H), 0.75 (t, *J* = 7.2 Hz, 3H).

**^13^C NMR (101 MHz, CDCl_3_)** δ 171.0, 170.5, 169.0, 146.3, 138.1, 130.9, 128.7, 128.2, 128.0, 127.9, 127.8, 80.6, 65.0, 62.0, 61.6, 44.1, 38.0, 24.4, 14.1, 13.8, 13.5.

**HRMS (ESI)** calcd for C_25_H_29_NO_4_ [M+H]^+^: 408.2169, found: 408.2178.

**diethyl 5-(4-butylphenyl)-2-phenyl-2,4-dihydro-3*H*-pyrrole-3,3-dicarboxylate (3ae)**

61.5 mg, 73% (eluent: PE/EA 20/1);

Appearance: yellow oil;

**^1^H NMR (400 MHz, CDCl_3_)** δ 7.86 (d, *J* = 8.2 Hz, 2H), 7.29 – 7.23 (m, 7H), 6.29 (s, 1H), 4.37 – 4.19 (m, 2H), 4.16 – 4.07 (m, 1H), 3.69-3.65 (m, 1H), 3.47 – 3.38 (m, 2H), 2.66 (t, *J* = 7.7 Hz, 2H), 1.68 – 1.57 (m, 2H), 1.42 – 1.31 (m, 2H), 1.29 (t, *J* = 7.1 Hz, 3H), 0.93 (t, *J* = 7.4 Hz, 3H), 0.82 (t, *J* = 7.2 Hz, 3H).

**^13^C NMR (101 MHz, CDCl_3_)** δ 194.8, 169.6, 168.1, 150.8, 143.7, 138.0, 129.6 (overlapped), 129.4, 128.5, 128.0, 127.7, 126.9, 62.2, 62.0, 59.9, 49.4, 38.7, 35.8, 32.9, 22.4, 13.9, 13.8.

**HRMS (ESI)** calcd for C_26_H_31_NO_4_ [M+Na]^+^: 444.2145, found: 444.2154.

**diethyl 5-(4-pentylphenyl)-2-phenyl-2,4-dihydro-3*H*-pyrrole-3,3-dicarboxylate (3af)**

54.0 mg, 62% (eluent: PE/EA 20/1);

Appearance: yellow oil;

**^1^H NMR (400 MHz, CDCl_3_)** δ 7.78 (d, *J* = 8.2 Hz, 2H), 7.25 – 7.13 (m, 7H), 6.21 (s, 1H), 4.25 – 4.11 (m, 2H), 4.09 – 4.01 (m, 1H), 3.67 – 3.57 (m, 1H), 3.40 – 3.29 (m, 2H), 2.58 (t, *J* = 7.7 Hz, 2H), 1.60 – 1.53 (m, 2H), 1.30 – 1.14 (m, 7H), 0.82 (t, *J* = 6.9 Hz, 3H), 0.75 (t, *J* = 7.1 Hz, 3H).

**^13^C NMR (101 MHz, CDCl_3_)** δ 171.0, 170.5, 169.0, 146.5, 138.1, 130.8, 128.7, 128.2, 128.0, 127.9, 127.8, 80.6, 65.0, 62.0, 61.6, 44.1, 35.9, 31.5, 31.0, 22.6, 14.1, 13.4.

**HRMS (ESI)** calcd for C_27_H_33_NO_4_ [M+H]^+^: 436.2482, found: 436.2492.

**diethyl 5-(4-fluorophenyl)-2-phenyl-2,4-dihydro-3*H*-pyrrole-3,3-dicarboxylate (3ag)**

46.0 mg, 60% (eluent: PE/EA 20/1);

Appearance: yellow oil;

**^1^H NMR (400 MHz, CDCl_3_)** δ 8.01 – 7.93 (m, 2H), 7.31 – 7.26 (m, 5H), 7.16 (t, *J* = 8.7 Hz, 2H), 6.30 (s, 1H), 4.41 – 4.20 (m, 2H), 4.17 – 4.09 (m, 1H), 3.77 – 3.66 (m, 1H), 3.49 – 3.37 (m, 2H), 1.32 (t, *J* = 7.1 Hz, 3H), 0.84 (t, *J* = 7.1 Hz, 3H).

**^13^C NMR (101 MHz, CDCl_3_)** δ 170.8, 169.4, 168.9, 164.6 (d, *J* = 251.7 Hz), 137.9, 130.1 (d, *J* = 8.6 Hz), 129.6 (d, *J* = 3.3 Hz), 128.2, 128.0, 127.9, 115.7 (d, *J* = 21.8 Hz), 80.7, 65.1, 62.1, 61.7, 44.2, 14.1, 13.4.

**^19^F NMR (376 MHz, CDCl_3_)** δ -108.73.

**HRMS (ESI)** calcd for C_22_H_22_FNO_4_ [M+H]^+^: 384.1606, found: 384.1613.

**diethyl 5-(4-chlorophenyl)-2-phenyl-2,4-dihydro-3*H*-pyrrole-3,3-dicarboxylate (3ah)**

44.7 mg, 56% (eluent: PE/EA 20/1);

Appearance: yellow oil;

**^1^H NMR (400 MHz, CDCl_3_)** δ 7.80 (d, *J* = 6.5 Hz, 2H), 7.35 (d, *J* = 6.5 Hz, 2H), 7.19 – 7.18 (m, 5H), 6.21 (s, 1H), 4.29 – 4.13 (m, 2H), 4.02 (d, *J* = 17.8 Hz, 1H), 3.66 – 3.57 (m, 1H), 3.37 – 3.30 (m, 2H), 1.22 (t, *J* = 7.2 Hz, 3H), 0.74 (t, *J* = 7.2 Hz, 3H).

**^13^C NMR (101 MHz, CDCl_3_)** δ 170.8, 169.6, 168.8, 137.9, 137.3, 131.8, 129.3, 128.9, 128.1, 128.0, 127.9, 80.7, 65.0, 62.1, 61.7, 44.1, 14.0, 13.4.

**HRMS (ESI)** calcd for C_22_H_22_ClNO_4_ [M+H]^+^: 400.1310, found: 400.1312.

**diethyl 2-phenyl-5-(4-(trifluoromethyl)phenyl)-2,4-dihydro-3*H*-pyrrole-3,3-dicarboxylate (3ai)**

39.8 mg, 46% (eluent: PE/EA 20/1);

Appearance: yellow oil;

**^1^H NMR (400 MHz,** **CDCl_3_)** δ 7.99 (d, *J* = 8.1 Hz, 2H), 7.64 (d, *J* = 8.2 Hz, 2H), 7.26 – 7.15 (m, 5H), 6.25 (s, 1H), 4.32 – 4.14 (m, 2H), 4.11 – 4.00 (m, 1H), 3.68 – 3.57 (m, 1H), 3.42 – 3.29 (m, 2H), 1.23 (t, *J* = 7.1 Hz, 3H), 0.74 (t, *J* = 7.2 Hz, 3H).

**^13^C NMR (101 MHz, CDCl_3_)** δ 170.71, 169.55, 168.78, 137.63, 136.47, 132.70 (q, *J* = 32.6 Hz), 128.30, 128.14, 128.05, 127.94, 125.59 (q, *J* = 3.8 Hz), 123.92 (q, *J* = 272.5 Hz), 80.85, 65.01, 62.20, 61.74, 44.20, 14.04, 13.40.

**^19^F NMR (376 MHz, CDCl_3_)** δ -62.86.

**HRMS (ESI)** calcd for C_23_H_22_F_3_NO_4_ [M+H]^+^: 434.1574, found: 434.1582.

**diethyl 2-phenyl-5-(*m*-tolyl)-2,4-dihydro-3*H*-pyrrole-3,3-dicarboxylate (3aj)**

31.8 mg, 42% (eluent: PE/EA 20/1);

Appearance: yellow solid;

Melting point: 57.1 – 59.3 °C;

**^1^H NMR (400 MHz, CDCl_3_)** δ 7.74 (s, 1H), 7.63 (d, *J* = 7.2 Hz, 1H), 7.29 – 7.15 (m, 7H), 6.22 (s, 1H), 4.32 – 4.12 (m, 2H), 4.10 – 4.01 (m, 1H), 3.68 – 3.56 (m, 1H), 3.41 – 3.29 (m, 2H), 2.33 (s, 3H), 1.22 (t, *J* = 7.1 Hz, 3H), 0.75 (t, *J* = 7.1 Hz, 3H).

**^13^C NMR (101 MHz, CDCl_3_)** δ 171.0, 170.8, 168.9, 138.4, 138.0, 133.2, 131.9, 128.5, 128.2, 128.0, 127.8, 125.2, 80.7, 64.9, 62.1, 61.6, 44.2, 21.3, 14.1, 13.4.

**HRMS (ESI)** calcd for C_23_H_25_NO_4_ [M+H]^+^: 380.1856, found: 380.1863.

**diethyl 5-(3-fluorophenyl)-2-phenyl-2,4-dihydro-3*H*-pyrrole-3,3-dicarboxylate (3ak)**

36.8 mg, 48% (eluent: PE/EA 20/1);

Appearance: yellow oil;

**^1^H NMR (400 MHz, CDCl_3_)** δ 7.69 (d, *J* = 8.5 Hz, 2H), 7.47 – 7.38 (m, 1H), 7.32 – 7.20 (m, 6H), 6.30 (s, 1H), 4.39 – 4.17

(m, 2H), 4.11 (d, *J* = 17.8 Hz, 1H), 3.78 – 3.62 (m, 1H), 3.46 – 3.37 (m, 2H), 1.30 (t, *J* = 7.1 Hz, 3H), 0.81 (t, *J* = 7.1 Hz, 3H).

**^13^C NMR (101 MHz, CDCl_3_)** δ 170.78, 169.62 (d, *J* = 2.7 Hz), 168.82, 162.85 (d, *J* = 246.8 Hz), 137.76, 135.48 (d, *J* = 7.7 Hz), 130.20 (d, *J* = 8.0 Hz), 128.15, 128.02, 127.96, 123.77 (d, *J* = 3.0 Hz), 118.12 (d, *J* = 21.3 Hz), 114.63 (d, *J* = 22.7 Hz), 80.69, 64.98, 62.15, 61.69, 44.19, 14.06, 13.42.

**^19^F NMR (376 MHz, CDCl_3_)** δ -112.44.

**HRMS (ESI)** calcd for C_22_H_22_FNO_4_ [M+H]^+^: 384.1606, found: 384.1609.

**diethyl 2-phenyl-5-(3-(trifluoromethyl)phenyl)-2,4-dihydro-3*H*-pyrrole-3,3-dicarboxylate (3al)**

62.4 mg, 72% (eluent: PE/EA 20/1);

Appearance: yellow oil;

**^1^H NMR (400 MHz, CDCl_3_)** δ 8.21 (s, 1H), 8.12 (d, *J* = 7.8 Hz, 1H), 7.75 (d, *J* = 8.0 Hz, 1H), 7.59 (t, *J* = 7.8 Hz, 1H), 7.30 – 7.24 (m, 5H), 6.32 (s, 1H), 4.42 – 4.20 (m, 2H), 4.16 (d, *J* = 17.9 Hz, 1H), 3.80 – 3.61 (m, 1H), 3.49 – 3.38 (m, 2H), 1.31 (t, *J* = 7.2 Hz, 3H), 0.82 (t, *J* = 7.2 Hz, 3H).

**^13^C NMR (101 MHz, CDCl_3_)** δ 170.72, 169.43, 168.78, 137.61, 134.02, 131.28 (q, *J* = 32.8 Hz), 131.15, 129.20, 128.15, 128.06, 127.70 (q, *J* = 3.2 Hz), 124.80 (q, *J* = 3.7 Hz), 123.92 (q, *J* = 271.6 Hz), 80.79, 65.00, 62.22, 61.75, 44.14, 14.05, 13.41.

**^19^F NMR (376 MHz, CDCl_3_)** δ -62.68.

**HRMS (ESI)** calcd for C_23_H_22_F_3_NO_4_ [M+H]^+^: 434.1574, found: 434.1581.

**diethyl 5-(2-methoxyphenyl)-2-phenyl-2,4-dihydro-3*H*-pyrrole-3,3-dicarboxylate (3am)**

33.2 mg, 42% (eluent: PE/EA 20/1);

Appearance: yellow oil;

**^1^H NMR (400 MHz, CDCl_3_)** δ 8.01 (d, *J* = 9.5 Hz, 1H), 7.50 – 7.35 (m, 1H), 7.33 – 7.20 (m, 5H), 7.05 – 6.93 (m, 2H), 6.19 (s, 1H), 4.38 – 4.12 (m, 3H), 3.91 (s, 3H), 3.76 – 3.64 (m, 1H), 3.53 (d, *J* = 18.6 Hz, 1H), 3.49 – 3.37 (m, 1H), 1.28 (t, *J* = 7.1 Hz, 3H), 0.83 (t, *J* = 7.2 Hz, 3H).

**^13^C NMR (101 MHz, CDCl_3_)** δ 171.3, 171.0, 169.1, 158.5, 138.2, 132.1, 130.6, 128.2, 127.9, 127.7, 123.1, 120.8, 111.3, 79.0, 65.3, 61.9, 61.4, 55.5, 47.3, 14.1, 13.5.

**HRMS (ESI)** calcd for C_23_H_25_NO_5_ [M+H]^+^: 396.1805, found: 396.1810.

**3.2 Photocatalytic [4+2] cyclization to access 1-tetralones**

An oven-dried Schlenk tube (10 mL) equipped with a stirring bar was charged with alkyl bromide **1** (0.2 mmol) and *f*ac-Ir(ppy)_3_ (2 mol%). The tube was connected to a vacuum line where it was evacuated and back-filled with Ar for three times. Next, vinyl azide **2** (0.4 mmol, 2.0 equiv.), AcOH (0.2 mmol, 1.0 equiv.), and dry CH_2_Cl_2_ (4 mL) were added under Ar flow. The tube was placed approximately 2 cm from 30 W blue LEDs, and then stirred at room temperature for 9 h. After completion, the reaction was quenched with H_2_O (6 mL) and extracted with EtOAc. The organic layer was washed with saturated brine and dried over Na_2_SO_4_. The solvent was removed under reduced pressure, and the resulting residue was purified by column chromatography on silica gel to afford the desired product.

**diethyl 7-methyl-4-oxo-1-phenyl-3,4-dihydronaphthalene-2,2(1*H*)-dicarboxylate (4aa)**

55.5 mg, 73% (eluent: PE/EA 20/1);

Appearance: yellow oil;

**^1^H NMR (400 MHz, CDCl_3_)** δ 7.98 (d, *J* = 8.0 Hz, 1H), 7.29 – 7.21 (m, 3H), 7.16 (d, *J* = 8.1 Hz, 1H), 7.05 – 6.98 (m, 3H), 5.17 (s, 1H), 4.17 – 4.00 (m, 4H), 3.30 (d, *J* = 18.1 Hz, 1H), 3.21 (d, *J* = 18.1 Hz, 1H), 2.31 (s, 3H), 1.17 (t, *J* = 7.2 Hz, 3H), 1.10 (t, *J* = 7.1 Hz, 3H).

**^13^C NMR (101 MHz, CDCl_3_)** δ 194.7, 169.6, 168.1, 146.0, 143.7, 138.0, 130.2, 129.6, 129.2, 128.7, 128.6, 127.7, 126.9, 62.2, 62.0, 59.8, 49.3, 38.7, 21.8, 13.9, 13.8.

**HRMS (ESI)** calcd for C_23_H_24_O_5_ [M+H]^+^: 381.1697, found: 381.1700.

**diethyl 7-methyl-4-oxo-1-(*p*-tolyl)-3,4-dihydronaphthalene-2,2(1*H*)-dicarboxylate (4ca)**

59.1 mg, 75% (eluent: PE/EA 20/1);

Appearance: yellow oil;

**^1^H NMR (400 MHz, CDCl_3_)** δ 7.97 (d, *J* = 8.1 Hz, 1H), 7.15 (d, *J* = 8.1 Hz, 1H), 7.08 – 7.00 (m, 3H), 6.89 (d, *J* = 7.7 Hz, 2H), 5.13 (s, 1H), 4.15 – 4.01 (m, 4H), 3.29 (d, *J* = 18.1 Hz, 1H), 3.19 (d, *J* = 18.1 Hz, 1H), 2.30 (s, 3H), 2.28 (s, 3H), 1.20 (t, *J* = 7.2 Hz, 3H), 1.10 (t, *J* = 7.1 Hz, 3H).

**^13^C NMR (101 MHz, CDCl_3_)** δ 194.8, 169.7, 168.1, 146.0, 144.0, 137.5, 134.9, 130.2, 129.4, 129.3, 129.2, 128.6, 126.8, 62.2, 61.9, 59.8, 49.0, 38.7, 21.8, 21.0, 13.9, 13.8.

**HRMS (ESI)** calcd for C_24_H_26_O_5_ [M+H]^+^: 395.1853, found: 395.1861.

**diethyl 1-(4-(*tert-*butyl)phenyl)-7-methyl-4-oxo-3,4-dihydronaphthalene-2,2(1*H*)-dicarboxylate (4da)**

39.2 mg, 45% (eluent: PE/EA 20/1);

Appearance: yellow oil;

**^1^H NMR (400 MHz, CDCl_3_)** δ 7.97 (d, *J* = 8.1 Hz, 1H), 7.24 (d, *J* = 8.3 Hz, 2H), 7.15 (d, *J* = 8.1 Hz, 1H), 7.04 (s, 1H), 6.92 (d, *J* = 8.0 Hz, 2H), 5.14 (s, 1H), 4.17 – 3.98 (m, 4H), 3.29 (d, *J* = 18.0 Hz, 1H), 3.19 (d, *J* = 18.1 Hz, 1H), 2.31 (s, 3H), 1.26 (s, 9H), 1.13 (t, *J* = 7.3 Hz, 3H), 1.09 (t, *J* = 7.5 Hz, 3H).

**^13^C NMR (101 MHz, CDCl_3_)** δ 194.93, 169.68, 168.18, 150.51, 145.94, 143.97, 134.74, 130.26, 129.21, 128.65, 126.84, 125.43, 62.23, 61.93, 59.91, 49.03, 38.75, 34.45, 31.27, 21.86, 13.85, 13.78.

**HRMS (ESI)** calcd for C_27_H_32_O_5_ [M+H]^+^: 437.2323, found: 437.2331.

**diethyl 1-(4-fluorophenyl)-7-methyl-4-oxo-3,4-dihydronaphthalene-2,2(1*H*)-dicarboxylate (4ea)**

53.3 mg, 67% (eluent: PE/EA 20/1);

Appearance: yellow oil;

**^1^H NMR (400 MHz, CDCl_3_)** δ 7.90 (d, *J* = 8.1 Hz, 1H), 7.10 (d, *J* = 8.1 Hz, 1H), 6.95 – 6.84 (m, 5H), 5.08 (s, 1H), 4.10 – 3.94 (m, 4H), 3.15 (s, 2H), 2.25 (s, 3H), 1.12 (t, *J* = 7.1 Hz, 3H), 1.03 (t, *J* = 7.1 Hz, 3H).

**^13^C NMR (101 MHz, CDCl_3_)** δ 194.5, 169.5, 168.0, 162.2 (d, *J* = 247.5 Hz), 146.2, 143.5, 133.8 (d, *J* = 3.4 Hz), 131.2 (d, *J* = 8.1 Hz), 130.2, 129.1, 128.9, 127.0, 115.5 (d, *J* = 21.5 Hz), 62.3, 62.1, 59.7, 48.5, 38.6, 21.8, 13.9, 13.8.

**^19^F NMR (376 MHz, CDCl_3_)** δ -114.37.

**HRMS (ESI)** calcd for C_23_H_23_FO_5_ [M+H]^+^: 399.1602, found: 399.1608.

**diethyl 1-(4-chlorophenyl)-7-methyl-4-oxo-3,4-dihydronaphthalene-2,2(1*H*)-dicarboxylate (4fa)**

38.9 mg, 47% (eluent: PE/EA 20/1);

Appearance: yellow solid.

Melting point: 149.6 – 152.9 °C.

**^1^H NMR (400 MHz, CDCl_3_)** δ 7.91 (d, *J* = 8.0 Hz, 1H), 7.17 – 7.08 (m, 3H), 6.99 (s, 1H), 6.94 (s, 1H), 6.81 – 6.74 (m, 1H), 5.07 (s, 1H), 4.09 – 3.97 (m, 4H), 3.16 (s, 2H), 2.26 (s, 3H), 1.15 (t, *J* = 7.2 Hz, 3H), 1.03 (t, *J* = 7.1 Hz, 3H).

**^13^C NMR (101 MHz, CDCl_3_)** δ 194.30, 169.32, 167.89, 146.22, 142.89, 140.13, 134.47, 130.20, 129.82, 129.77, 129.14, 129.06, 127.93, 127.72, 127.07, 62.41, 62.24, 59.65, 48.90, 38.63, 21.85, 13.94, 13.77.

**HRMS (ESI)** calcd for C_23_H_23_ClO_5_ [M+H]^+^: 415.1307, found: 415.1315.

**diethyl 1-(4-bromophenyl)-7-methyl-4-oxo-3,4-dihydronaphthalene-2,2(1*H*)-dicarboxylate (4ga)**

56.8 mg, 62% (eluent: PE/EA 20/1);

Appearance: yellow solid;

Melting point: 150.2 – 150.4 °C;

**^1^H NMR (400 MHz, CDCl_3_)** δ 7.98 (d, *J* = 8.0 Hz, 1H), 7.38 (d, *J* = 8.0 Hz, 2H), 7.18 (d, *J* = 8.1 Hz, 1H), 6.99 (s, 1H), 6.90 (d, *J* = 8.1 Hz, 2H), 5.13 (s, 1H), 4.16 – 4.03 (m, 4H), 3.23 (s, 2H), 2.32 (s, 3H), 1.20 (t, *J* = 7.2 Hz, 3H), 1.10 (t, *J* = 7.2 Hz, 3H).

**^13^C NMR (101 MHz, CDCl_3_)** δ 194.3, 169.3, 167.9, 146.2, 143.1, 137.1, 131.7, 131.3, 130.1, 129.1, 129.0, 127.0, 121.9, 62.4, 62.2, 59.6, 48.7, 38.6, 21.8, 13.9, 13.8.

**HRMS (ESI)** calcd for C_23_H_23_BrO_5_ [M+H]^+^: 459.0802, found: 459.0806.

**diethyl 1-(4-cyanophenyl)-7-methyl-4-oxo-3,4-dihydronaphthalene-2,2(1*H*)-dicarboxylate (4ha)**

58.3 mg, 72% (eluent: PE/EA 20/1);

Appearance: yellow solid.

Melting point: 110.0 – 110.5 °C.

**^1^H NMR (400 MHz, CDCl_3_)** δ 8.00 (d, *J* = 8.1 Hz, 1H), δ 7.57 (d, *J* = 8.4 Hz, 2H), 7.21 (d, *J* = 8.2 Hz, 1H), 7.16 (d, *J* = 8.4 Hz, 2H), 6.98 (s, 1H), 5.23 (s, 1H), 4.16 – 4.04 (m, 4H), 3.31 – 3.24 (m, 1H), 3.15 (d, *J* = 18.1 Hz, 1H), 2.33 (s, 3H), 1.20 (t, *J* = 7.2 Hz, 3H), 1.11 (t, *J* = 7.1 Hz, 3H).

**^13^C NMR (101 MHz, CDCl_3_)** δ 193.8, 169.0, 167.7, 146.4, 143.6, 142.2, 132.3, 130.5, 130.1, 129.3, 129.2, 127.2, 118.3, 111.8, 62.6, 62.3, 59.6, 49.2, 38.6, 21.8, 13.9, 13.7.

**HRMS (ESI)** calcd for C_24_H_23_NO_5_ [M+H]^+^:406.1649, found: 406.1658.

**diethyl 1-(4-acetylphenyl)-7-methyl-4-oxo-3,4-dihydronaphthalene-2,2(1*H*)-dicarboxylate (4ia)**

59.1 mg, 70% (eluent: PE/EA 20/1);

Appearance: yellow solid;

Melting point: 77.8 – 79.9 °C;

**^1^H NMR (400 MHz, CDCl_3_)** δ 8.00 (d, *J* = 8.0 Hz, 1H), 7.85 (d, *J* = 8.5 Hz, 2H), 7.20 (d, *J* = 8.1 Hz, 1H), 7.13 (d, *J* = 8.3 Hz, 2H), 7.00 (s, 1H), 5.22 (s, 1H), 4.18 – 4.01 (m, 4H), 3.24 (s, 2H), 2.55 (s, 3H), 2.32 (s, 3H), 1.20 (t, *J* = 7.1 Hz, 3H), 1.11 (t, *J* = 7.1 Hz, 3H).

**^13^C NMR (101 MHz, CDCl_3_)** δ 197.5, 194.3, 169.3, 167.9, 146.3, 143.5, 142.9, 136.3, 130.1, 129.9, 129.2, 129.1, 128.6, 127.1, 62.5, 62.2, 59.6, 49.1, 38.7, 26.6, 21.8, 13.9, 13.8.

**HRMS (ESI)** calcd for C_25_H_26_O_6_ [M+Na]^+^: 445.1622, found: 445.1628.

**diethyl 1-(3-fluorophenyl)-7-methyl-4-oxo-3,4-dihydronaphthalene-2,2(1*H*)-dicarboxylate (4ja)**

46.2 mg, 58% (eluent: PE/EA 20/1);

Appearance: yellow oil;

**^1^H NMR (400 MHz, CDCl_3_)** δ 7.98 (d, *J* = 8.1 Hz, 1H), 7.23 – 7.18 (m, 2H), 7.02 (s, 1H), 6.96 – 6.92 (m, 1H), 6.79 (d, *J* = 7.8 Hz, 1H), 6.74 (d, *J* = 9.9 Hz, 1H), 5.16 (s, 1H), 4.13 – 4.07 (m, 4H), 3.24 (s, 2H), 2.33 (s, 3H), 1.21 (t, *J* = 7.2 Hz, 3H), 1.10 (t, *J* = 7.2 Hz, 3H).

**^13^C NMR (101 MHz, CDCl_3_)** δ 194.3, 169.4, 167.9, 162.7 (d, *J* = 247.1 Hz), 146.2, 143.0, 140.6 (d, *J* = 6.7 Hz), 130.2, 130.06 (d, *J* = 8.3 Hz), 129.1, 129.0, 127.1, 125.2 (d, *J* = 3.0 Hz), 116.7 (d, *J* = 22.1 Hz), 114.7 (d, *J* = 21.0 Hz), 62.4, 62.2, 59.7, 48.9, 38.7, 21.8, 13.9, 13.8.

**^19^F NMR (376 MHz, CDCl_3_)** δ -112.30.

**HRMS (ESI)** calcd for C_23_H_23_FO_5_ [M+H]^+^: 399.1602, found: 399.1609.

**diethyl 1-(3-chlorophenyl)-7-methyl-4-oxo-3,4-dihydronaphthalene-2,2(1*H*)-dicarboxylate (4ka)**

49.7 mg, 60% (eluent: PE/EA 20/1);

Appearance: yellow solid;

Melting point: 123.7 – 127.8 °C;

**^1^H NMR (400 MHz, CDCl_3_)** δ 7.99 (d, *J* = 10.2 Hz, 1H), 7.25 – 7.13 (m, 3H), 7.06 (s, 1H), 7.01 (s, 1H), 6.85 (d, *J* = 5.8 Hz, 1H), 5.14 (s, 1H), 4.17 – 4.03 (m, 4H), 3.23 (s, 2H), 2.33 (s, 3H), 1.22 (t, *J* = 7.2 Hz, 3H), 1.10 (t, *J* = 7.1 Hz, 3H).

**^13^C NMR (101 MHz, CDCl_3_)** δ 194.3, 169.3, 167.9, 146.2, 142.9, 140.2, 134.5, 130.2, 129.8, 129.8, 129.2, 129.1, 127.9, 127.7, 127.1, 62.4, 62.2, 59.7, 48.9, 38.6, 21.8, 13.9, 13.8.

**HRMS (ESI)** calcd for C_23_H_23_ClO_5_ [M+H]^+^: 415.1307, found: 415.1316.

**diethyl 1-(3-bromophenyl)-7-methyl-4-oxo-3,4-dihydronaphthalene-2,2(1*H*)-dicarboxylate (4la)**

66.0 mg, 72% (eluent: PE/EA 20/1);

Appearance: yellow solid;

Melting point: 118.9 – 122.5 °C;

**^1^H NMR (400 MHz, CDCl_3_)** δ 7.98 (d, *J* = 8.0 Hz, 1H), 7.37 (d, *J* = 9.7 Hz, 1H), 7.24 (s, 1H), 7.19 (d, *J* = 8.1 Hz, 1H), 7.13 –7.09 (m, 1H), 7.01 (s, 1H), 6.87 (d, *J* = 9.5 Hz, 1H), 5.13 (s, 1H), 4.15 – 4.07 (m, 4H), 3.23 (s, 2H), 2.33 (s, 3H), 1.23 (t, *J* = 7.1 Hz, 3H), 1.10 (t, *J* = 6.9 Hz, 3H).

**^13^C NMR (101 MHz, CDCl_3_)** δ 194.2, 169.3, 167.9, 146.2, 142.8, 140.8, 132.7, 130.8, 130.2, 130.1, 129.2, 129.1, 128.2, 127.1, 122.7, 62.4, 62.3, 59.7, 48.9, 38.6, 21.8, 14.0, 13.8.

**HRMS (ESI)** calcd for C_23_H_23_BrO_5_ [M+H]^+^: 459.0802, found: 459.0806.

**diethyl 1-(3-methoxyphenyl)-7-methyl-4-oxo-3,4-dihydronaphthalene-2,2(1*H*)-dicarboxylate (4ma)**

52.5 mg, 64% (eluent: PE/EA 20/1);

Appearance: yellow oil;

**^1^H NMR (400 MHz, CDCl_3_)** δ 7.96 (d, *J* = 8.0 Hz, 1H), 7.15 (d, *J* = 6.3 Hz, 2H), 7.03 (s, 1H), 6.76 (d, *J* = 8.2 Hz, 1H), 6.62 – 6.48 (m, 2H), 5.13 (s, 1H), 4.17 – 4.00 (m, 4H), 3.71 (s, 3H), 3.32 (d, *J* = 17.9 Hz, 1H), 3.20 (d, *J* = 18.1 Hz, 1H), 2.31 (s, 3H), 1.19 (t, *J* = 7.0 Hz, 3H), 1.09 (t, *J* = 7.4 Hz, 3H).

**^13^C NMR (101 MHz, CDCl_3_)** δ 194.7, 169.6, 168.1, 159.6, 146.0, 143.6, 139.5, 130.2, 129.5, 129.2, 128.8, 126.9, 121.9, 116.1, 112.4, 62.3, 62.0, 59.8, 55.1, 49.3, 38.8, 21.8, 13.9, 13.8.

**HRMS (ESI)** calcd for C_24_H_26_O_6_ [M+H]^+^: 411.1802, found: 411.1805.

**diethyl 7-methyl-4-oxo-1-(*o*-tolyl)-3,4-dihydronaphthalene-2,2(1*H*)-dicarboxylate (4na)**

36.2 mg, 46% (eluent: PE/EA 20/1);

Appearance: yellow oil;

**^1^H NMR (400 MHz, CDCl_3_)** δ 7.95 (d, *J* = 8.1 Hz, 1H), 7.20 (d, *J* = 7.4 Hz, 1H), 7.14 – 7.08 (m, 2H), 7.03– 7.01 (m, 1H), 6.92 (s, 1H), 6.79 (d, *J* = 7.5 Hz, 1H), 5.57 (s, 1H), 4.19 – 4.06 (m, 2H), 4.02 – 3.94 (m, 1H), 3.91 – 3.83 (m, 1H), 3.59 (d, *J* = 18.2 Hz, 1H), 3.28 – 3.19 (m, 1H), 2.59 (s, 3H), 2.29 (s, 3H), 1.13 (t, *J* = 7.3 Hz, 3H), 1.01 (t, *J* = 7.3 Hz, 3H).

**^13^C NMR (101 MHz, CDCl_3_)** δ 194.7, 170.1, 168.4, 145.9, 145.0, 136.9, 136.5, 131.0, 130.1, 129.9, 128.9, 128.5, 127.3, 126.8, 126.2, 62.3, 61.9, 59.0, 43.4, 39.1, 21.8, 20.3, 13.8, 13.6.

**HRMS (ESI)** calcd for C_24_H_26_O_5_ [M+H]^+^: 395.1853, found: 395.1860.

**diethyl 1-(2-fluorophenyl)-7-methyl-4-oxo-3,4-dihydronaphthalene-2,2(1*H*)-dicarboxylate (4oa)**

34.2 mg, 43% (eluent: PE/EA 20/1);

Appearance: yellow oil;

**^1^H NMR (400 MHz, CDCl_3_)** δ 7.90 (d, *J* = 8.1 Hz, 1H), 7.21– 7.20 (m, 1H), 7.16 (d, *J* = 8.1 Hz, 1H), 7.02 – 6.87 (m, 3H), 6.79– 6.75 (m, 1H), 5.45 (s, 1H), 4.11 – 3.94 (m, 4H), 3.28 (d, *J* = 18.2 Hz, 1H), 3.14 (d, *J* = 18.2 Hz, 1H), 2.24 (s, 3H), 1.12 (t, *J* = 7.2 Hz, 3H), 1.04 (t, *J* = 7.1 Hz, 3H).

**^13^C NMR (101 MHz, CDCl_3_)** δ 194.45, 169.61, 168.18, 160.67 (d, *J* = 247.2 Hz), 146.05, 143.22, 131.51 (d, *J* = 3.3 Hz), 130.08, 129.43 (d, J = 8.7 Hz), 129.39, 128.87, 126.96, 125.46 (d, *J* = 14.2 Hz), 124.27 (d, *J* = 3.6 Hz), 115.69 (d, *J* = 23.1 Hz), 62.20, 59.04, 41.70, 41.68, 39.26, 21.81, 13.77, 13.70.

**^19^F NMR (376 MHz, CDCl_3_)** δ -115.49.

**HRMS (ESI)** calcd for C_23_H_23_FO_5_ [M+H]^+^: 399.1602, found: 399.1607.

**diethyl 1-(2-chlorophenyl)-7-methyl-4-oxo-3,4-dihydronaphthalene-2,2(1*H*)-dicarboxylate (4pa)**

56.3 mg, 68% (eluent: PE/EA 20/1);

Appearance: yellow solid;

Melting point: 142.3 – 143.3 °C;

**^1^H NMR (400 MHz, CDCl_3_)** δ 7.99 (d, *J* = 8.0 Hz, 1H), 7.23 – 7.14 (m, 3H), 7.09 – 6.99 (m, 2H), 6.86 (d, *J* = 7.5 Hz, 1H), 5.15 (s, 1H), 4.17 – 4.04 (m, 4H), 3.24 (s, 2H), 2.32 (s, 3H), 1.22 (t, *J* = 7.2 Hz, 3H), 1.10 (t, *J* = 7.1 Hz, 3H).

**^13^C NMR (101 MHz, CDCl_3_)** δ 194.23, 169.28, 167.87, 146.18, 142.89, 140.15, 134.45, 130.19, 129.82, 129.76, 129.15, 129.04, 127.91, 127.73, 127.04, 62.37, 62.21, 59.64, 48.90, 38.64, 21.81, 13.92, 13.76.

**HRMS (ESI)** calcd for C_23_H_23_ClO_5_ [M+H]^+^: 415.1307, found: 415.1316.

**diethyl 1-(3,5-dimethylphenyl)-7-methyl-4-oxo-3,4-dihydronaphthalene-2,2(1*H*)-dicarboxylate (4qa)**

41.6 mg, 51% (eluent: PE/EA 20/1);

Appearance: yellow oil;

**^1^H NMR (400 MHz, CDCl_3_)** δ 7.97 (d, *J* = 8.0 Hz, 1H), 7.16 (d, *J* = 9.7 Hz, 1H), 7.03 (s, 1H), 6.86 (s, 1H), 6.60 (s, 2H), 5.09 (s, 1H), 4.15 – 4.02 (m, 4H), 3.32 (d, *J* = 18.0 Hz, 1H), 3.19 (d, *J* = 18.1 Hz, 1H), 2.31 (s, 3H), 2.21 (s, 6H), 1.21 (t, *J* = 7.1 Hz, 3H), 1.10 (t, *J* = 7.1 Hz, 3H).

**^13^C NMR (101 MHz, CDCl_3_)** δ 195.0, 169.7, 168.2, 146.0, 144.0, 138.0, 137.8, 130.2, 129.4, 129.2, 128.6, 127.4, 126.8, 62.2, 61.9, 59.8, 49.3, 38.8, 21.8, 21.3, 13.9, 13.8.

**HRMS (ESI)** calcd for C_25_H_28_O_5_ [M+H]^+^: 409.2010, found: 409.2018.

**diethyl 7-methoxy-4-oxo-1-phenyl-3,4-dihydronaphthalene-2,2(1*H*)-dicarboxylate (4ab)**

46.0 mg, 58% (eluent: PE/EA 20/1);

Appearance: yellow oil;

**^1^H NMR (400 MHz, CDCl_3_)** δ 7.70 (d, *J* = 7.9 Hz, 1H), 7.35– 7.33 (m, 1H), 7.28 – 7.16 (m, 3H), 7.05 – 7.03 (m, 3H), 5.49 (s, 1H), 4.18 – 3.96 (m, 4H), 3.72 (s, 3H), 3.31 (d, *J* = 18.2 Hz, 1H), 3.21 (d, *J* = 18.2 Hz, 1H), 1.19 (t, *J* = 7.1 Hz, 3H), 1.08 (t, *J* = 7.1 Hz, 3H).

**^13^C NMR (101 MHz, CDCl_3_)** δ 195.5, 169.7, 168.2, 156.6, 137.2, 132.6, 132.4, 129.5, 128.3, 128.1, 127.4, 118.4, 116.2, 62.2, 62.0, 59.7, 56.0, 43.2, 38.7, 13.9, 13.8.

**HRMS (ESI)** calcd for C_23_H_24_O_6_ [M+H]^+^: 397.1646, found: 397.1648.

**diethyl 7-ethyl-4-oxo-1-phenyl-3,4-dihydronaphthalene-2,2(1H)-dicarboxylate (4ac)**

55.2 mg, 70% (eluent: PE/EA 20/1);

Appearance: yellow oil;

**^1^H NMR (400 MHz, CDCl_3_)** δ 8.00 (d, *J* = 8.0 Hz, 1H), 7.26 – 7.16 (m, 4H), 7.06 – 6.96 (m, 3H), 5.18 (s, 1H), 4.15 – 4.01 (m, 4H), 3.28 (d, *J* = 18.0 Hz, 1H), 3.21 (d, *J* = 18.1 Hz, 1H), 2.61 (q, *J* = 7.6 Hz, 2H), 1.18 (t, *J* = 7.6 Hz, 3H), 1.17 (t, *J* = 7.1 Hz, 3H), 1.08 (t, *J* = 7.1 Hz, 3H).

**^13^C NMR (101 MHz, CDCl_3_)** δ 194.8, 169.6, 168.1, 152.0, 143.8, 138.0, 129.6, 129.5, 129.0, 128.5, 127.7, 127.5, 126.9, 62.2, 62.0, 59.9, 49.4, 38.7, 29.0, 14.7, 13.9, 13.8.

**HRMS (ESI)** calcd for C_24_H_26_O_5_ [M+H]^+^: 395.1853, found: 395.1855.

**diethyl 4-oxo-1-phenyl-7-propyl-3,4-dihydronaphthalene-2,2(1*H*)-dicarboxylate (4ad)**

38.4 mg, 47% (eluent: PE/EA 20/1);

Appearance: yellow oil;

**^1^H NMR (400 MHz, CDCl_3_)** δ 7.92 (d, *J* = 8.1 Hz, 1H), 7.21 – 7.14 (m, 3H), 7.10 (d, *J* = 8.1 Hz, 1H), 6.98 – 6.91 (m, 3H), 5.11 (s, 1H), 4.09 – 3.94 (m, 4H), 3.21 (d, *J* = 18.1 Hz, 1H), 3.14 (d, *J* = 18.1 Hz, 1H), 2.49 – 2.45 (m, 2H), 1.54– 1.49 (m, 2H), 1.10 (t, *J* = 7.1 Hz, 3H), 1.01 (t, *J* = 7.1 Hz, 3H), 0.81 (t, *J* = 7.3 Hz, 3H).

**^13^C NMR (101 MHz, CDCl_3_)** δ 194.83, 169.60, 168.12, 150.58, 143.66, 138.03, 129.67, 129.58, 129.48, 128.54, 128.00, 127.67, 126.86, 62.23, 61.98, 59.88, 49.42, 38.73, 38.10, 23.89, 13.88, 13.79, 13.76.

**HRMS (ESI)** calcd for C_25_H_28_O_5_ [M+H]^+^: 409.2010, found: 409.2013.

**diethyl 7-butyl-4-oxo-1-phenyl-3,4-dihydronaphthalene-2,2(1*H*)-dicarboxylate (4ae)**

38.0 mg, 45% (eluent: PE/EA 20/1);

Appearance: yellow oil;

**^1^H NMR (400 MHz, CDCl_3_)** δ 7.92 (d, *J* = 8.1 Hz, 1H), 7.21 – 7.14 (m, 3H), 7.13 – 7.08 (m, 1H), 6.98 – 6.91 (m, 3H), 5.11 (s, 1H), 4.08 – 3.94 (m, 4H), 3.20 (d, *J* = 18.1 Hz, 1H), 3.13 (d, *J* = 16.7 Hz, 1H), 2.49 (t, *J* = 8.7 Hz, 2H), 1.53 – 1.41 (m, 2H), 1.28 – 1.17 (m, 2H), 1.10 (t, *J* = 7.1 Hz, 3H), 1.01 (t, *J* = 7.1 Hz, 3H), 0.80 (t, *J* = 7.3 Hz, 3H).

**^13^C NMR (101 MHz, CDCl_3_)** δ 194.8, 169.6, 168.1, 150.8, 143.7, 138.0, 129.6 (overlapped), 129.5, 128.5, 127.9, 127.7, 126.9, 62.2, 62.0, 59.9, 49.4, 38.7, 35.8, 32.9, 22.4, 13.9, 13.8.

**HRMS (ESI)** calcd for C_26_H_30_O_5_ [M+H]^+^: 423.2166, found: 423.2168.

**diethyl 4-oxo-7-pentyl-1-phenyl-3,4-dihydronaphthalene-2,2(1*H*)-dicarboxylate (4af)**

34.9 mg, 40% (eluent: PE/EA 20/1);

Appearance: yellow oil;

**^1^H NMR (400 MHz, CDCl_3_)** δ 7.92 (d, *J* = 8.0 Hz, 1H), 7.22 – 7.14 (m, 3H), 7.13 – 7.07 (m, 1H), 6.98 – 6.91 (m, 3H), 5.11 (s, 1H), 4.11 – 3.91 (m, 4H), 3.21 (d, *J* = 18.0 Hz, 1H), 3.14 (d, *J* = 18.0 Hz, 1H), 2.48 (t, *J* = 8.8Hz, 2H), 1.55 – 1.40 (m, 2H), 1.23 – 1.15 (m, 4H), 1.10 (t, *J* = 7.1 Hz, 3H), 1.01 (t, *J* = 7.1 Hz, 3H), 0.77 (t, *J* = 6.8 Hz, 3H).

**^13^C NMR (101 MHz, CDCl_3_)** δ 194.8, 169.6, 168.1, 150.9, 143.7, 138.0, 129.6 (overlapped), 129.4, 128.5, 127.9, 127.7, 126.9, 62.2, 62.0, 59.9, 49.4, 38.7, 36.1, 31.5, 30.4, 22.4, 14.0, 13.9, 13.8.

**HRMS (ESI)** calcd for C_27_H_32_O_5_ [M+H]^+^: 437.2323, found: 437.2327.

**diethyl 7-fluoro-4-oxo-1-phenyl-3,4-dihydronaphthalene-2,2(1*H*)-dicarboxylate (4ag)**

46.1 mg, 60% (eluent: PE/EA 20/1);

Appearance: yellow oil;

**^1^H NMR (400 MHz, CDCl_3_)** δ 8.09 – 8.00 (m, 1H), 7.22 – 7.15 (m, 3H), 7.01 – 6.90 (m, 3H), 6.88 – 6.79 (m, 1H), 5.11 (s, 1H), 4.10 – 3.94 (m, 4H), 3.24 (d, *J* = 18.1 Hz, 1H), 3.15 (d, *J* = 18.1 Hz, 1H), 1.10 (t, *J* = 7.2 Hz, 3H), 1.04 (t, *J* = 7.1 Hz, 3H).

**^13^C NMR (101 MHz, CDCl_3_)** δ 193.5, 169.5, 167.8, 166.6 (d, *J* = 257.5 Hz), 146.9 (d, *J* = 9.3 Hz), 137.1, 130.0 (d, *J* = 10.0 Hz), 129.5, 128.7, 128.1 (d, J = 2.0 Hz), 128.0, 116.3 (d, *J* = 21.8 Hz), 115.6 (d, *J* = 22.2 Hz), 62.4, 62.1, 59.7, 49.3, 38.6, 13.9, 13.8.

**^19^F NMR (376 MHz, CDCl_3_)** δ -102.10.

**HRMS (ESI)** calcd for C_22_H_21_FO_5_ [M+Na]^+^: 407.1205, found: 407.1208.

**diethyl 7-chloro-4-oxo-1-phenyl-3,4-dihydronaphthalene-2,2(1*H*)-dicarboxylate (4ah)**

38.4 mg, 48% (eluent: PE/EA 20/1);

Appearance: yellow oil;

**^1^H NMR (400 MHz, CDCl_3_)** δ 8.03 (d, *J* = 8.4 Hz, 1H), 7.34 (d, *J* = 8.4 Hz, 1H), 7.29 – 7.21 (m, 4H), 7.03 – 6.95 (m, 2H), 5.17 (s, 1H), 4.19 – 4.00 (m, 4H), 3.31 (d, *J* = 18.2 Hz, 1H), 3.23 (d, *J* = 18.3 Hz, 1H), 1.18 (t, *J* = 7.1 Hz, 3H), 1.12 (t, *J* = 7.0 Hz, 3H).

**^13^C NMR (101 MHz, CDCl_3_)** δ 193.93, 169.40, 167.73, 145.31, 141.24, 137.10, 129.84, 129.82, 129.55, 128.75, 128.45, 128.24, 128.03, 62.46, 62.16, 59.63, 49.11, 38.66, 13.88, 13.80.

**HRMS (ESI)** calcd for C_22_H_21_ClO_5_ [M+H]^+^: 401.1150, found: 401.1156.

**diethyl 6-methyl-4-oxo-1-phenyl-3,4-dihydronaphthalene-2,2(1*H*)-dicarboxylate (4aj)**

**diethyl 8-methoxy-4-oxo-1-phenyl-3,4-dihydronaphthalene-2,2(*1H*)-dicarboxylate (4aj')**

55.4 mg, 70% (eluent: PE/EA 20/1);

**4aj**:**4aj'** = 1.2:1;

Appearance: yellow oil;

**^1^H NMR (400 MHz, CDCl_3_)** δ 7.92 (d, *J* = 7.8 Hz, 1H), 7.82 (s, 1.2H), 7.30 (d, *J* = 7.4 Hz, 1H), 7.25 (d, *J* = 5.9 Hz, 1.2H), 7.22 (d, *J* = 7.6 Hz, 1H), 7.19 – 7.11 (m, 6.6H), 7.05 (d, *J* = 7.9 Hz, 1.2H), 7.00 – 6.88 (m, 4.4H), 5.20 (s, 1H), 5.12 (s, 1.2H), 4.11 – 3.91 (m, 8.8H), 3.28 – 3.10 (m, 4.4H), 2.30 (s, 3.6H), 2.13 (s, 3H), 1.11 (t, *J* = 7.1 Hz, 3H), 1.10 (t, *J* = 7.1 Hz, 3.6H), 1.03 (t, *J* = 7.1 Hz, 3.6H), 1.00 (t, *J* = 7.1 Hz, 3H).

**^13^C NMR (101 MHz, CDCl_3_)** δ 195.6, 195.3, 169.60, 169.58, 168.2, 168.1, 141.9, 140.9, 138.1, 137.4, 137.0, 136.7, 136.1, 136.0, 131.8, 131.2, 129.9, 129.8, 129.6, 128.6, 128.5, 127.71, 127.66, 127.3, 126.8, 124.8, 62.3 (overlapped), 62.1, 62.0, 59.9, 59.8, 49.0, 46.6, 38.8, 38.5, 21.0, 19.3, 13.90, 13.88, 13.8, 13.7.

**HRMS (ESI)** calcd for C_23_H_24_O_5_ [M+H]^+^: 381.1697, found: 381.1704.

**diethyl 5-chloro-4-oxo-1-phenyl-3,4-dihydronaphthalene-2,2(1*H*)-dicarboxylate (4an)**

26.4 mg, 33% (eluent: PE/EA 20/1);

Appearance: yellow oil;

**^1^H NMR (400 MHz, CDCl_3_)** δ 7.33 – 7.27 (m, 2H), 7.20 – 7.16 (m, 3H), 7.13 – 7.09 (m, 1H), 6.98 – 6.93 (m, 2H), 5.15 (s, 1H), 4.10 – 3.95 (m, 4H), 3.28 (d, *J* = 18.2 Hz, 1H), 3.20 (d, *J* = 18.2 Hz, 1H), 1.11 (t, *J* = 7.2 Hz, 3H), 1.05 (t, *J* = 7.1 Hz, 3H).

**^13^C NMR (101 MHz, CDCl_3_)** δ 193.9, 169.4, 167.7, 145.3, 141.3, 137.1, 129.8 (overlapped), 129.5, 128.8, 128.5, 128.3, 128.0, 62.5, 62.2, 59.6, 49.1, 38.7, 13.9, 13.8.

**HRMS (ESI)** calcd for C_22_H_21_ClO_5_ [M+H]^+^: 401.1150, found: 401.1159.

**diethyl 4-oxo-7-phenyl-4,7-dihydrobenzo[b]thiophene-6,6(5*H*) -dicarboxylate (4ao)**

35.0 mg, 47% (eluent: PE/EA 20/1);

Appearance: yellow oil;

**^1^H NMR (400 MHz, CDCl_3_)** δ 7.41 (d, *J* = 4.8 Hz, 1H), 7.32 – 7.24 (m, 3H), 7.18 (d, *J* = 5.0 Hz, 1H), 7.10 – 7.02 (m, 2H), 5.42 (s, 1H), 4.25 – 3.95 (m, 4H), 3.26 (d, *J* = 17.9 Hz, 1H), 3.16 (d, *J* = 17.9 Hz, 1H), 1.15 (t, *J* = 7.4 Hz, 6H).

**^13^C NMR (101 MHz, CDCl_3_)** δ 189.3, 169.2, 167.5, 156.1, 137.0, 136.5, 128.9, 128.8, 128.3, 125.5, 124.1, 62.5, 62.1, 61.9, 46.1, 38.6, 13.8.

**HRMS (ESI)** calcd for C_20_H_20_S [M+H]^+^: 373.1104, found: 373.1110

# 4. Synthetic applications of the reaction

**4.1** **Scale-up reaction**

An oven-dried Schlenk tube (25 mL) equipped with a stirring bar was charged with alkyl bromide **1a** (0.328 g, 1 mmol), K_2_CO_3_ (0.207 g, 1.5 mmol), and Ru(bpy)_3_(PF_6_)_2_ (0.172 g, 0.02 mmol). The tube was connected to a vacuum line where it was evacuated and back-filled with Ar for three times. Next, vinyl azide **2a** (0.397 g, 2.5 mmol) and dry CH_2_Cl_2_ (10 mL) were added under Ar flow. The tube was placed approximately 2 cm from 30 W blue LEDs, and then stirred at room temperature for 9 h. After completion, the reaction was quenched with H_2_O (30 mL) and extracted with EtOAc. The organic layer was washed with saturated brine and dried over Na_2_SO_4_. The solvent was removed under reduced pressure, and the resulting residue was purified by column chromatography on silica gel with petroleum ether/ethyl acetate (40:1) as eluent to afford the desired product **3aa** (0.193 g, 51%).

**4.2 Product derivatizations**

**(a) The synthesis of diethyl 2-phenyl-5-(*p*-tolyl)-1-(2,2,2-trifluoroacetyl)-1,2-dihydro-3*H*-**

**pyrrole-3,3-dicarboxylate(Wang et al., 2011)**

An oven-dried Schlenk tube (10 mL) equipped with a stirring bar was charged with **3aa** (37.9 mg, 0.1 mmol). The tube was connected to a vacuum line where it was evacuated and back-filled with Ar for three times. Anhydrous THF (2 mL) was added under Ar flow. Next, the solution was stirred in the ice bath, and (CF_3_CO)_2_O (13.9 μL, 0.1 mmol) was added under Ar flow. The resulting mixture was stirred at 0 °C for 12 h. After completion, the reaction was quenched with a saturated solution of NaHCO_3_ and extracted with EtOAc. The organic layer was washed with saturated brine and dried over Na_2_SO_4_. The solvent was removed under reduced pressure, and then the resulting residue was purified by column chromatography on silica gel with petroleum ether/ethyl acetate (20:1 ~ 10:1) as eluent to afford the desired product **5** (36.7 mg, 89%). Appearance: colorless oil. **^1^H NMR (400 MHz, CDCl_3_)** δ 7.37 – 7.28 (m, 7H), 7.25 – 7.22 (m, 2H), 6.27 (s, 1H), 5.96 (s, 1H), 4.39 – 4.20 (m, 2H), 3.80 – 3.60 (m, 2H), 2.40 (s, 3H), 1.30 (t, *J* = 7.0 Hz, 3H), 0.92 (t, *J* = 7.2 Hz, 3H). **^13^C NMR (101 MHz, CDCl_3_)** δ 167.7, 166.3, 155.6 (q, *J* = 38.6 Hz), 147.5, 139.8, 135.7, 129.2, 129.1, 128.4, 127.7, 127.1, 126.3, 115.8 (q, *J* = 288.7 Hz), 114.1, 62.8, 61.9, 21.4, 14.0, 13.5. **^19^F NMR (376 MHz, CDCl_3_)** δ -70.53. **HRMS (ESI)** calcd for C_25_H_24_F_3_NO_5_ [M+H]^+^: 476.1679, found: 476.1684.

**(b) The synthesis of diethyl 1-acetyl-2-phenyl-5-(*p*-tolyl)-1,2-dihydro-3*H*- pyrrole-3,3-dicarboxylate(Xu and Smith, 2021)**

An oven-dried Schlenk tube (10 mL) equipped with a stirring bar was charged with **3aa** (37.9 mg, 0.1 mmol). The tube was connected to a vacuum line where it was evacuated and back-filled with Ar for three times. Anhydrous CH_2_Cl_2_ (2 mL) and 2,6-lutidine (32.1 mg, 0.3 mmol) were added under Ar flow. Next, the solution was stirred in the ice bath, and acetyl chloride (11.8 mg, 0.15 mmol) was added under Ar flow. The resulting mixture was stirred at 0 °C for 15 min. Then, the resulting mixture was stirred at 40 °C for 9 h. After completion, the reaction was quenched with water and extracted with EtOAc. The organic layer was washed with saturated brine and dried over Na_2_SO_4_. The solvent was removed under reduced pressure, and then the resulting residue was purified by column chromatography on silica gel with petroleum ether/ethyl acetate (40:1 ~ 20:1) as eluent to afford the desired product **6** (40.7 mg, 90%). Appearance: colorless oil. **^1^H NMR (400 MHz, CDCl_3_)** δ 7.43 – 7.34 (m, 4H), 7.34 – 7.27 (m, 3H), 7.25 – 7.18 (m, 2H), 6.19 (s, 1H), 5.58 (s, 1H), 4.40 – 4.19 (m, 2H), 3.77 – 3.55 (m, 2H), 2.39 (s, 3H), 1.88 (s, 3H), 1.30 (t, *J* = 7.2 Hz, 3H), 0.86 (t, *J* = 6.9 Hz, 3H). **^13^C NMR (101 MHz, CDCl_3_)** δ 168.9, 168.5, 167.4, 147.5, 139.2, 136.8, 129.6, 129.1, 128.6, 128.4, 127.5, 127.0, 110.2, 69.2, 62.4, 61.6, 21.4, 14.1, 13.5. **HRMS (ESI)** calcd for C_25_H_27_NO_5_ [M+H]^+^: 422.1962, found: 422.1969.

**(c) The synthesis of diethyl 4,4-dichloro-2-phenyl-5-(*p*-tolyl)-2,4-dihydro-3*H*-pyrrole-3,3-dicarboxylate(Zhan et al., 2015)**

An oven-dried Schlenk tube (10 mL) equipped with a stirring bar was charged with **3aa** (40.0 mg, 0.1 mmol) and *N*-chlorosuccinimide (NCS) (80.1 mg, 0.6 mmol). The tube was connected to a vacuum line where it was evacuated and back-filled with Ar for three times. Next, CCl_4_ (1 mL) was added under Ar flow. The reaction mixture was stirred at 80 °C for 8 h. After completion, the reaction was quenched with water and extracted with EtOAc. The organic layer was washed with saturated brine and dried over Na_2_SO_4_. The solvent was removed under reduced pressure, and then the resulting residue was purified by column chromatography on silica gel with petroleum ether/ethyl acetate (40:1 ~ 20:1) as eluent to afford the desired product **8** (39.3 mg, 88%). Appearance: white solid. Melting point: 152.2-152.4 °C. **^1^H NMR (400 MHz, CDCl_3_)** δ 8.06 (d, *J* = 7.8 Hz, 2H), 7.62 (d, *J* = 7.4 Hz, 2H), 7.38 – 7.27 (m, 5H), 6.05 (s, 1H), 4.52 – 4.39 (m, 2H), 3.74 – 3.63 (m, 1H), 3.58 – 3.45 (m, 1H), 2.44 (s, 3H), 1.40 (t, *J* = 7.3 Hz, 3H), 0.80 (t, *J* = 7.4 Hz, 3H). **^13^C NMR (101 MHz, CDCl_3_)** δ 168.9, 165.8, 164.3, 141.8, 136.6, 129.1, 128.9, 128.5, 128.1, 128.0, 127.0, 89.0, 79.2, 73.5, 62.5, 61.9, 21.6, 14.0, 13.3. **HRMS (ESI)** calcd for C_23_H_23_Cl_2_NO_4_ [M+H]^+^: 448.1077, found: 448.1085.

**(d) The synthesis of diethyl 3-([1,1'-biphenyl]-4-yl)-5-phenyl-7a-(*p*-tolyl)-7,7a-dihydropyrrolo [1,2-*d*][1,2,4]oxadiazole-6,6(5*H*)-dicarboxylate(Wang et al., 2019)**

An oven-dried Schlenk tube (10 mL) equipped with a stirring bar was charged with **3aa** (37.9 mg, 0.1 mmol) and *N*-hydroxy-[1,1'-biphenyl]-4-carbimidoyl chloride (60.0 mg, 0.22 mmol). The tube was connected to a vacuum line where it was evacuated and back-filled with Ar for three times. Next, CH_2_Cl_2_ (2 mL) and Et_3_N (35.0 μL, 0.25 mmol) were added in turn under Ar flow. The reaction mixture was stirred at room temperature for 8 h. After completion, the reaction was quenched with a saturated solution of NaHCO_3_ and extracted with EtOAc. The organic layer was washed with saturated brine and dried over Na_2_SO_4_. The solvent was removed under reduced pressure, and then the resulting residue was purified by column chromatography on silica gel with petroleum ether/ethyl acetate (40:1 ~ 20:1) as eluent to afford the desired product **7** (47.0 mg, 92%). Appearance: white solid. Melting point: 193.1-194.2 °C. **^1^H NMR (400 MHz, CDCl_3_)** δ 7.77 (d, *J* = 7.8 Hz, 2H), 7.61 – 7.54 (m, 6H), 7.45 – 7.42 (m, 2H), 7.38 – 7.36 (m, 1H), 7.32 – 7.26 (m, 3H), 7.25 – 7.23 (m, 3H), 7.22 – 7.20 (s, 1H), 5.62 (s, 1H), 4.41 – 4.25 (m, 2H), 3.86 – 3.75 (m, 1H), 3.73 – 3.63 (m, 1H), 3.56

(d, *J* = 15.2 Hz, 1H), 3.50 (d, *J* = 15.2 Hz, 1H), 2.39 (s, 3H), 1.32 (t, *J* = 7.2 Hz, 3H), 0.95 (t, *J* = 7.2 Hz, 3H). **^13^C NMR (101 MHz, CDCl_3_)** δ 169.4, 166.4, 160.1, 143.7, 140.3, 138.7, 138.2, 137.4, 129.4, 128.9, 128.5, 128.3, 127.9, 127.8, 127.5, 127.2, 125.6, 125.3, 107.8, 71.4, 66.4, 62.7, 61.8, 42.8, 21.3, 14.0, 13.6. **HRMS (ESI)** calcd for C_36_H_34_N_2_O_5_ [M+H]^+^: 575.2540, found: 575.2546.

**(e) The synthesis of ethyl 4-methyl-2-phenyl-5-(*p*-tolyl)-1*H*-pyrrole-3-carboxylate(Zhu and Chiba, 2016)**

An oven-dried round-bottom flask (25 mL) equipped with a stirring bar was charged with **3aa** (75.8 mg, 0.2 mmol) and LiCl (25.4 mg, 0.6 mmol). The tube was connected to a vacuum line where it was evacuated and back-filled with Ar for three times. Next, DMSO (6 mL) and H_2_O (14.4 μL, 0.8 mmol) were added in turn under Ar flow. The reaction mixture was reflux stirred at 180 ℃ for 8 h. After completion, the reaction mixture was extracted with EtOAc. The organic layer was washed with saturated brine and dried over Na_2_SO_4_. The solvent was removed under reduced pressure, and then the resulting residue was purified by column chromatography on silica gel with petroleum ether/ethyl acetate (40:1 ~ 20:1) as eluent to afford the desired product **9** (41.5 mg, 65%). Appearance: yellow oil. **^1^H NMR (400 MHz, CDCl_3_)** δ 8.22 (s, 1H), 7.58 – 7.50 (m, 2H), 7.43 – 7.32 (m, 5H), 7.24 (s, 2H), 4.21 (q, *J* = 7.1 Hz, 2H), 2.42 (s, 3H), 2.39 (s, 3H), 1.19 (t, *J* = 7.1 Hz, 3H). **^13^C NMR (101 MHz, CDCl_3_)** δ 165.80, 136.91, 136.54, 132.90, 129.63, 129.59, 129.52, 128.98, 128.07, 128.02, 127.39, 118.57, 113.05, 59.52, 21.21, 14.14, 11.79. **HRMS (ESI)** calcd for C_21_H_21_NO_2_ [M+H]^+^: 320.1645, found: 320.1656.

**4.3 Late-stage functionalization**

An oven-dried Schlenk tube (10 mL) equipped with a stirring bar was charged with alkyl bromide **1a** (65.6 mg, 0.2 mmol), K_2_CO_3_ (41.5 mg, 0.3 mmol), and Ru(bpy)_3_(PF_6_)_2_ (3.44 mg, 2 mol%). The tube was connected to a vacuum line where it was evacuated and back-filled with Ar for three times. Next, vinyl azide **2p** (160.6 mg, 0.5 mmol) and dry CH_2_Cl_2_ (2 mL) were added under Ar flow. The tube was placed approximately 2 cm from 30 W blue LEDs, and then stirred at room temperature for 9 h. After completion, the reaction was quenched with H_2_O (2.5 mL) and extracted with EtOAc. The organic layer was washed with saturated brine and dried over Na_2_SO_4_. The solvent was removed under reduced pressure, and then the resulting residue was purified by column chromatography on silica gel to afford the desired product **3ap** (77.9 mg, 72%). Appearance: white solid. Melting point: 184.6-185.7 °C. **^1^H NMR (400 MHz, CDCl_3_)** δ 7.74 (d, *J* = 7.5 Hz, 1H), 7.70– 7.67 (m, 1H), 7.38 (d, *J* = 8.2 Hz, 1H), 7.27 – 7.22 (m, 5H), 6.28 (s, 1H), 4.36 – 4.19 (m, 2H), 4.15 – 4.09 (m, 1H), 3.74 – 3.65 (m, 1H), 3.47 – 3.38 (m, 2H), 3.02 – 2.93 (m, 2H), 2.57 – 2.43 (m, 2H), 2.41 – 2.30 (m, 1H), 2.23 – 1.95 (m, 4H), 1.70 – 1.43 (m, 6H), 1.29 (t, *J* = 7.1 Hz, 3H), 0.93 (s, 3H), 0.83 (t, *J* = 7.1 Hz, 3H). **^13^C NMR (101 MHz, CDCl_3_)** δ 195.1, 171.0, 170.5, 168.9, 143.3, 138.1, 137.0, 130.8, 128.5, 128.2, 127.9, 127.8, 125.6, 125.4, 80.6, 65.0, 62.0, 61.6, 50.5, 47.9, 44.6, 44.1, 38.0, 35.9, 31.6, 26.4, 25.7, 21.6, 14.1, 13.8, 13.4. **HRMS (ESI)** calcd for C_34_H_39_NO_5_ [M+H]^+^: 542.2901, found: 542.2897.

**5. Mechanistic Studies**

**5.1 Cyclic voltammetry test**

Cyclic voltammetry test was performed in a three-electrode cell under argon at room temperature. All cyclic voltammograms were measured using Ag/Ag^+^ (0.01 M AgNO_3_ in MeCN) reference electrode, platinum (Pt) wire counter electrode, and a glassy carbon working electrode. The conditions of the experiments were as follows: testing compounds are in a solution of 0.1 M hexafluorophosphate (*^n^*Bu_4_NPF_6)_ in MeCN at a scan rate of 50 mV/s; Prior to each measurement, solutions were purged with argon (Ar) for 10 minutes to ensure the oxygen-free conditions.

Measuring the Fc/Fc^+^ redox couple afforded *E*_1/2_ = +0.06 V *vs.* Ag/Ag^+^ under our experimental conditions. The obtained value was referenced to Ag/Ag^+^ and converted to SCE by subtracting 0.36 V, providing a value of +0.42 V for the Fc/Fc^+^ couple.(Nicewicz et al., 2015) The reduction half-peak potential of **1a** and diethyl 2-bromomalonate in MeCN was measured as −1.63 V and −1.43 V (*vs.* Ag/Ag^+^), and correspondingly calculated to −1.27 V (*vs.* SCE) and −1.07 V (*vs.* SCE, nearly consistent with the literature −1.0 V *vs.* SCE),(Wang et al., 2016) respectively.

**Figure S1.** Cyclic voltammetry of **1a** (5 mM) in MeCN (*vs.* Ag/Ag^+^) with ^n^Bu_4_NPF_6_ (0.1 M) under argon at a glassy carbon electrode at a scan rate of 50 mV/s.

**Figure S2.** Cyclic voltammetry of diethyl 2-bromomalonate (5 mM) in MeCN (*vs.* Ag/Ag^+^) with ^n^Bu_4_NPF_6_ (0.1 M) under argon at a glassy carbon electrode at a scan rate of 50 mV/s.

**5.2 Radical trapping experiment**

**Trapping experiment with TEMPO**

An oven-dried Schlenk tube (10 mL) equipped with a stirring bar was charged with alkyl bromide **1a** (65.6 mg, 0.2 mmol), K_2_CO_3_(41.5 mg, 0.3 mmol), 2,2,6,6-tetramethyl-piperidinyloxyl (TEMPO) (93.8 mg, 0.6 mmol), and Ru(bpy)_3_(PF_6_)_2_ (3.44 mg, 2 mol%). The tube was connected to a vacuum line where it was evacuated and back-filled with Ar for three times. Next, vinyl azide **2a** (79.6 mg, 0.5 mmol) and dry CH_2_Cl_2_ (2 mL) were added under Ar flow. The tube was placed approximately 2 cm from 30 W blue LEDs, and then stirred at room temperature for 9 h. After completion, the corresponding **1a**-derived TEMPO-trapped adduct **TEMPO**-**1a**, **TEMPO-C**, and **TEMPO-C'** were detected through ESI-HRMS analysis, and the desired product **3aa** was not detected.

**Figure S3.** The ESI-HRMS spectrum of the corresponding **1a**-derived **TEMPO-1a**.

**Figure S4.** The ESI-HRMS spectrum of the corresponding TEMPO-trapped adduct **TEMPO-C**.

**Figure S5.** The ESI-HRMS spectrum of the corresponding TEMPO-trapped adduct **TEMPO-C'**.

**Trapping experiment with BHT**

An oven-dried Schlenk tube (10 mL) equipped with a stirring bar was charged with alkyl bromide **1a** (65.6 mg, 0.2 mmol), K_2_CO_3_ (41.5 mg, 0.3 mmol), *p*-dinitrobenzene (BHT) (108.1 mg, 0.6 mmol), and Ru(bpy)_3_(PF_6_)_2_ (3.44 mg, 2 mol%). The tube was connected to a vacuum line where it was evacuated and back-filled with Ar for three times. Next, vinyl azide **2a** (79.6 mg, 0.5 mmol) and dry CH_2_Cl_2_ (2 mL) were added under Ar flow. The tube was placed approximately 2 cm from 30 W blue LEDs, and then stirred at room temperature for 9 h. After completion, the yield of **3aa** declined to 40%.

**Trapping experiment with DPE**

An oven-dried Schlenk tube (10 mL) equipped with a stirring bar was charged with alkyl bromide **1a** (65.6 mg, 0.2 mmol), K_2_CO_3_ (41.5 mg, 0.3 mmol), and Ru(bpy)_3_(PF6)_2_ (3.4 mg, 2 mol%). The tube was connected to a vacuum line where it was evacuated and back-filled with Ar for three times. Next, vinyl azide **2a** (79.6 mg, 0.5 mmol), 1,1-diphenylethylene (DPE) (88 μL, 0.5 mmol), and dry CH_2_Cl_2_ (2 mL) were added under Ar flow. The tube was placed approximately 2 cm from 30 W blue LEDs, and then stirred at room temperature for 9 h. After completion, the addition products **3aa'** was captured by LC-MS analysis. Then, the reaction was quenched with H_2_O (4.5 mL) and extracted with EtOAc. The organic layer was washed with saturated brine and dried over Na_2_SO_4_. The solvent was removed under reduced pressure, and the resulting residue was purified by column chromatography on silica gel with petroleum ether/ethyl acetate (20:1~10:1) as eluent to afford the desired product **3aa'** (8.7 mg, 14%). Appearance: colorless oil. **^1^H NMR (400 MHz, CDCl_3_)** δ 7.37 – 7.35 (m, 3H), 7.26 – 7.14 (m, 12H), 6.59 (s, 1H), 4.01 – 3.84 (m, 4H), 3.37 (s, 2H), 1.16 (t, *J* = 7.3 Hz, 6H). **^13^C NMR (101 MHz, CDCl_3_)** δ 169.79, 142.63, 138.27, 138.06, 136.09, 130.17, 129.92, 128.15, 128.12, 127.99, 127.71, 127.47, 127.24, 126.98, 126.20, 61.47, 60.06, 42.82, 13.82. **HRMS (ESI)** calcd for C_28_H_29_O_4_ [M+H]^+^: 429.2060, found: 429.2052.

**5.3 Electron-transfer scavenging experiment**

ua

An oven-dried Schlenk tube (10 mL) equipped with a stirring bar was charged with alkyl bromide **1a** (65.6 mg, 0.2 mmol), K_2_CO_3_ (41.5 mg, 0.3 mmol), *p*-dinitrobenzene (**DNB**) (84 mg, 0.5 mmol), and Ru(bpy)_3_(PF_6_)_2_ (3.44 mg, 2 mol%). The tube was connected to a vacuum line where it was evacuated and back-filled with Ar for three times. Next, vinyl azide **2a** (79.6 mg, 0.5 mmol) and dry CH_2_Cl_2_ (2 mL) were added under Ar flow. The tube was placed approximately 2 cm from 30 W blue LEDs, and then stirred at room temperature for 9 h. After completion, the desired product **3aa** was not detected.

**5.4 Analysis of the key intermediate: 2*H*-azirine (2a')**

An oven-dried Schlenk tube (10 mL) equipped with a stirring bar was charged with K_2_CO_3_ (41.5 mg, 0.3 mmol) and Ru(bpy)_3_(PF_6_)_2_ (3.4 mg, 2 mol%). The tube was connected to a vacuum line where it was evacuated and back-filled with Ar for three times. Next, vinyl azide **2a** (79.6 mg, 0.5 mmol) and dry CH_2_Cl_2_ (2 mL) were added under Ar flow. The tube was placed approximately 2 cm from 30 W blue LEDs, and then stirred at room temperature for 9 h. After completion, the reaction was quenched with H_2_O (4.5 mL) and extracted with EtOAc. The organic layer was washed with saturated brine and dried over Na_2_SO_4_. The solvent was removed under reduced pressure, and then the resulting residue was purified by column chromatography on silica gel to afford the product **2a'** (24.9 mg, 38%). **Appearance:** colorless oil. **^1^H NMR (400 MHz, CDCl_3_)** δ 7.80 (d, *J* = 8.0 Hz, 2H), 7.37 (d, *J* = 7.9 Hz, 2H), 2.46 (s, 3H), 1.76 (s, 2H). **^13^C NMR (101 MHz, CDCl_3_)** δ 165.3, 143.8, 129.8, 129.6, 122.7, 21.9, 19.5.

An oven-dried Schlenk tube (10 mL) equipped with a stirring bar was charged with alkyl bromide **1a** (65.6 mg, 0.2 mmol), K_2_CO_3_ (41.5 mg, 0.3 mmol), and Ru(bpy)_3_(PF_6_)_2_ (3.4 mg, 2 mol%). The tube was connected to a vacuum line where it was evacuated and back-filled with Ar for three times. Next, **2a'** (65.5 mg, 0.5 mmol) and CH_2_Cl_2_ (2 mL) were added under Ar flow. The tube was placed approximately 2 cm from 30 W blue LEDs, and then stirred at room temperature for 9 h. After completion, the desired product **3aa** was not detected. The result suggested that 2*H*-azirine **2a'** was not the key intermediate in this cyclization reaction.

**5.5 ESI-HRMS analysis of the [3+2] cyclization reaction**

An oven-dried Schlenk tube (10 mL) equipped with a stirring bar was charged with alkyl bromide **1** (0.1 mmol), K_2_CO_3_ (0.15 mmol, 1.5 equiv.), and Ru(bpy)_3_(PF_6_)_2_ (2 mol%). The tube was connected to a vacuum line where it was evacuated and back-filled with Ar for three times. Next, vinyl azide **2** (0.25 mmol, 2.5 equiv.) and dry CH_2_Cl_2_ (2 mL) were added under Ar flow. The tube was placed approximately 2 cm from 30 W blue LEDs, and then stirred at room temperature for 5 h. The intermediate **I**, compound **C**•**H** and corresponding hydrolyzed **C•H**, compound **J•Br** and corresponding hydrolyzed **J•Br** were detected through ESI-HRMS analysis.


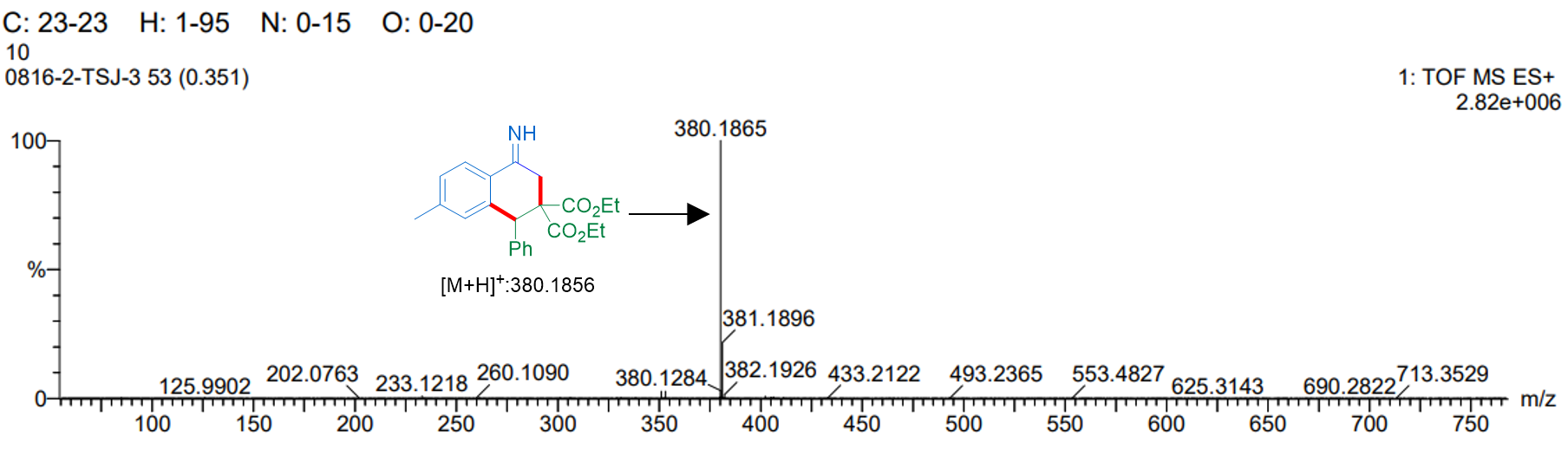


**Figure S6.** The ESI-HRMS spectrum of **I**


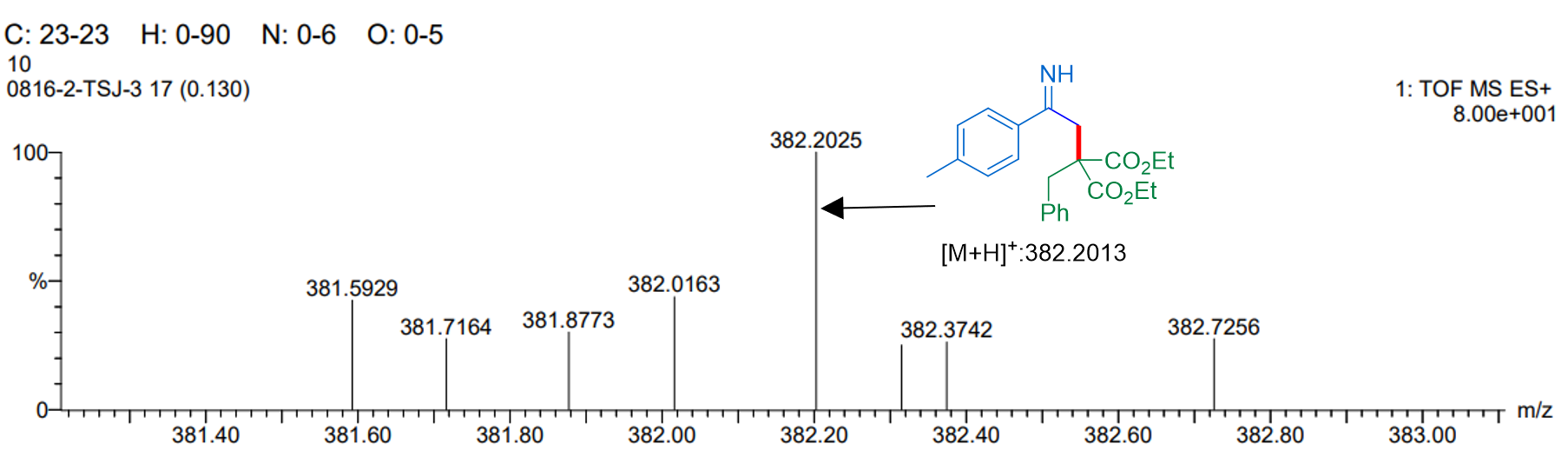


**Figure S7.** The ESI-HRMS spectrum of **C•H**


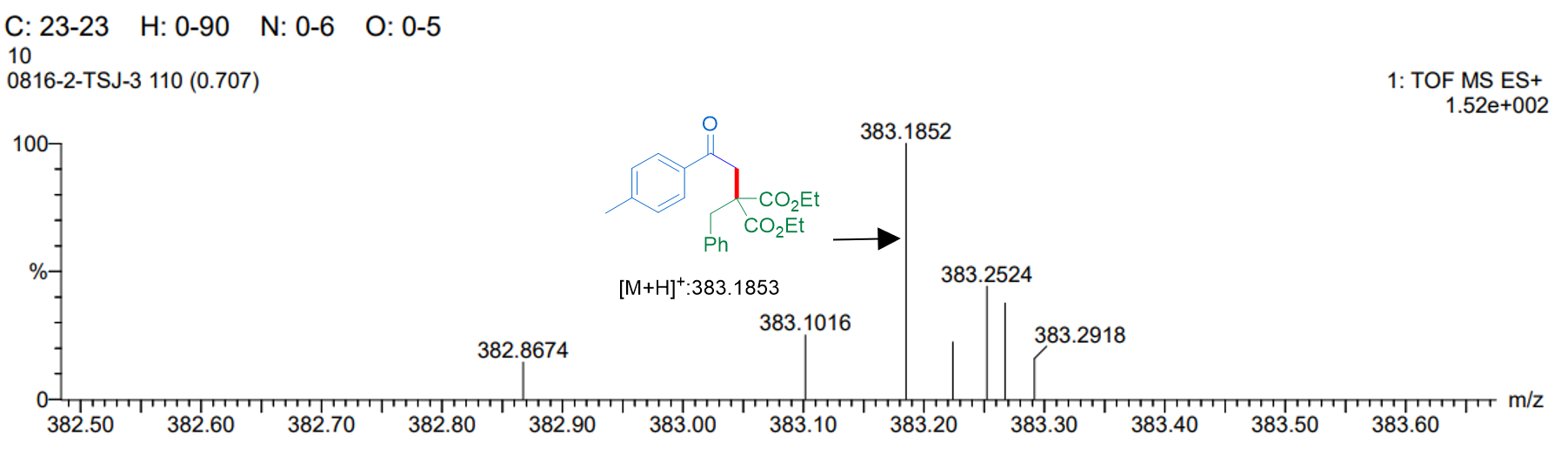


**Figure S8.** The ESI-HRMS spectrum of hydrolyzed **C•H**


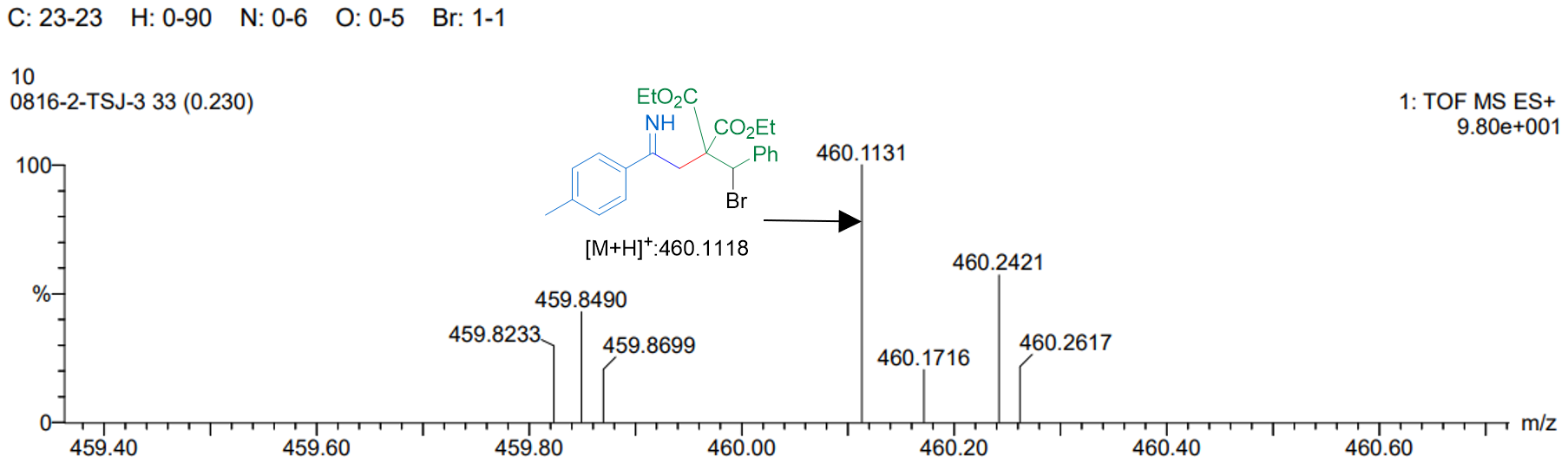


**Figure S9.** The ESI-HRMS spectrum of **J•Br**


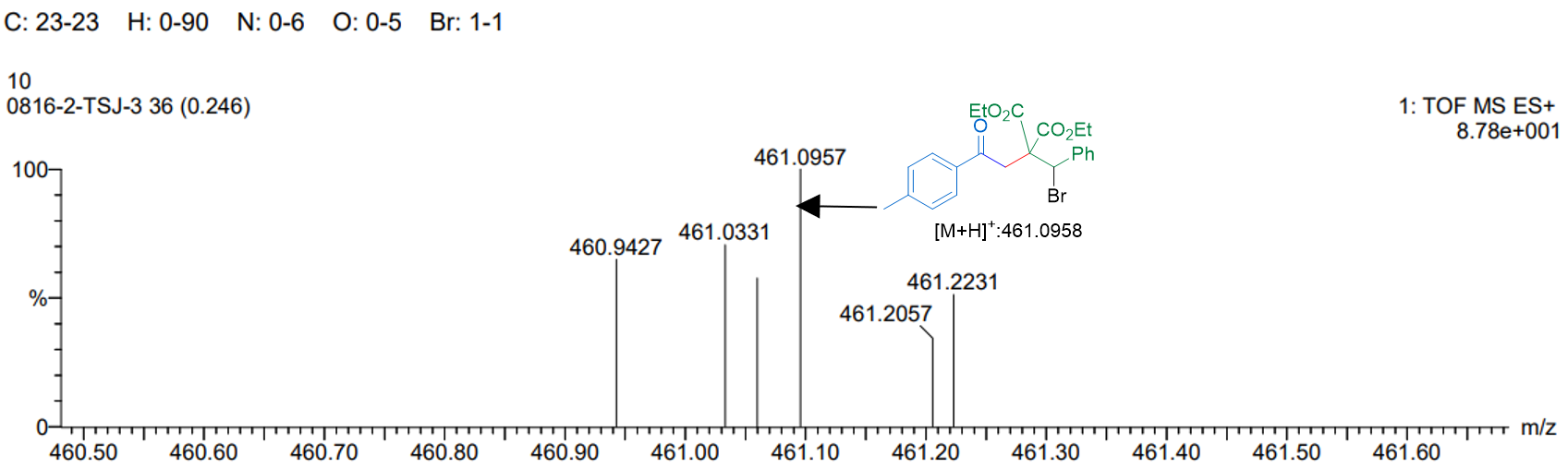


**Figure S10.** The ESI-HRMS spectrum of hydrolyzed **J•Br**

# 6. X-ray crystal data

**6.1 X-ray crystal data of 3ba**


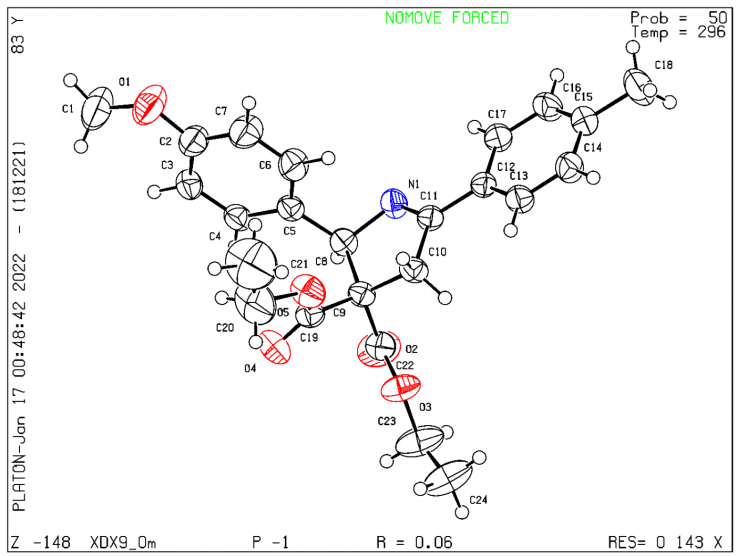


**Figure S11.** X-ray crystal structure of **3ba**

(The crystal was obtained by slow evaporation of the solution of CH_2_Cl_2_ and hexane) (**CCDC 2073209**):

| Bond precision: | C-C = 0.0026 Å | | Wavelength=0.71073 |
| --- | --- | --- | --- |
| Cell: | a=8.776 (2) | b=10.530 (3) | c = 13.052 (3) |
|  | alpha=71.261 (3) | beta=76.132 (3) | gamma=81.031 (3) |
| Temperature: | 296 K | |  |
|  | **Calculated** | | **Reported** |
| Volume | 1104.8 (5) | | 1104.9 (5) |
| Space group | P -1 | | P -1 |
| Hall group | -P 1 | | -P 1 |
| Moiety formula | C_24_H_27_NO_5_ | | ? |
| Sum formula | C_24_H_27_NO_5_ | | C_24_H_27_NO_5_ |
| Mr | 409.47 | | 409.46 |
| Dx, g cm^-3^ | 1.231 | | 1.231 |
| Z | 2 | | 2 |
| Mu (mm^-1^) | 0.086 | | 0.086 |
| F000 | 436.0 | | 436.0 |
| F000′ | 436.22 | |  |
| h, k, l_max_ | 11, 13, 16 | | 11, 13, 16 |
| Nref | 5079 | | 4993 |
| T_min_, T_max_ | 0.982, 0.985 | |  |
| T_min′_ | 0.982 | |  |
| Correction method = Not given |  | | |
| Data completeness = 0.983 | Theta(max) = 27.507 | |  |
| R(reflections) = 0.0560 (3855) | wR2(reflections) = 0.2031 (4993) | | |
| S = 1.171 | Npar = 275 | |  |

**6.2 X-ray crystal data of 4ga**


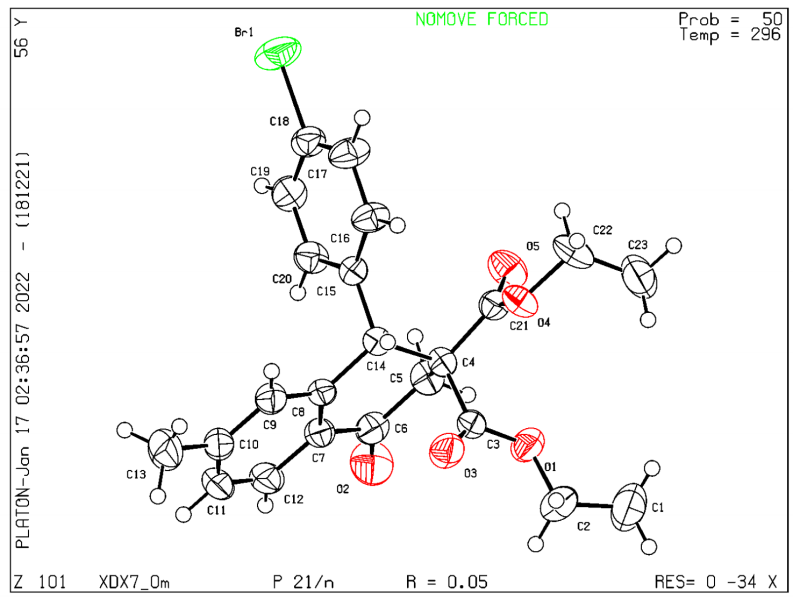


**Figure S12.** X-ray crystal structure of **4ga**

(The crystal was obtained by slow evaporation of the solution of CH_2_Cl_2_ and hexane) (**CCDC 2073208**):

| Bond precision: | C-C = 0.0043 Å | | Wavelength=0.71073 |
| --- | --- | --- | --- |
| Cell: | a=10.0907 (19) | b=19.254 (4) | c = 11.634 (2) |
|  | alpha=90 | beta=107.517 (3) | gamma=90 |
| Temperature: | 296 K | |  |
|  | **Calculated** | | **Reported** |
| Volume | 2155.5 (7) | | 2155.6 (7) |
| Space group | P 21/n | | P 21/n |
| Hall group | -P 2yn | | -P 2yn |
| Moiety formula | C_23_H_23_BrO_5_ | | ? |
| Sum formula | C_23_H_23_BrO_5_ | | C_23_H_23_BrO_5_ |
| Mr | 459.31 | | 459.32 |
| Dx, g cm^-3^ | 1.415 | | 1.415 |
| Z | 4 | | 4 |
| Mu (mm^-1^) | 1.938 | | 1.938 |
| F000 | 944.0 | | 944.0 |
| F000′ | 943.29 | |  |
| h, k, l_max_ | 13, 24, 15 | | 13, 24, 15 |
| Nref | 4920 | | 4881 |
| T_min_, T_max_ | 0.672, 0.706 | |  |
| T_min′_ | 0.659 | |  |
| Correction method = Not given |  | | |
| Data completeness = 0.992 | Theta(max) = 27.463 | |  |
| R(reflections) = 0.0502 (3116) | wR2(reflections) = 0.1548 (4881) | | |
| S = 0.955 | Npar = 265 | |  |

# 7. Supplementary references

Arceo, E., Montroni, E., and Melchiorre, P. (2014). Photo-organocatalysis of atom-transfer radical additions to alkenes. *Angew. Chem., Int. Ed. Engl.* 53, 12064-12068. doi:10.1002/anie.201406450

Jiao, M.J., Hu, Q., Hu, X.Q., and Xu, P.F. (2021). Visible-light-promoted multistep tandem reaction of vinyl azides toward the formation of 1-tetralones. *J. Org. Chem.* 86, 17156–17163. doi:10.1021/acs.joc.1c02261

Liu, Z., Liao, P., and Bi, X. (2014). General silver-catalyzed hydroazidation of terminal alkynes by combining TMS-N3 and H2O: synthesis of vinyl azides. *Org. Lett.* 16, 3668-3671. doi:10.1021/ol501661k

Nicewicz, D., Roth, H., and Romero, N. (2015). Experimental and calculated electrochemical potentials of common organic molecules for applications to single-electron redox chemistry. *Synlett* 27, 714-723. doi:10.1055/s-0035-1561297

Wang, D.S., Ye, Z.S., Chen, Q.A., Zhou, Y.G., Yu, C.B., Fan, H.J., et al. (2011). Highly enantioselective partial hydrogenation of simple pyrroles: a facile access to chiral 1-pyrrolines. *J. Am. Chem. Soc.* 133, 8866-8869. doi:10.1021/ja203190t

Wang, L., Huang, W., Li, R., Gehrig, D., Blom, P.W., Landfester, K., et al. (2016). Structural design principle of small-molecule organic semiconductors for metal-free, visible-light-promoted photocatalysis. *Angew. Chem., Int. Ed. Engl.* 55, 9783–9787. doi:10.1002/anie.201603789

Wang, Y., Ding, J., Zhao, J., Sun, W., Lian, C., Chen, C., et al. (2019). Iminyl radical-promoted imino sulfonylation, imino cyanogenation and imino thiocyanation of γ,δ-unsaturated oxime esters: synthesis of versatile functionalized pyrrolines. *Org. Chem. Front.* 6, 2240-2244. doi:10.1039/c9qo00421a

Xu, F., and Smith, M.W. (2021). A general approach to 2,2-disubstituted indoxyls: total synthesis of brevianamide A and trigonoliimine C. *Chem. Sci.* 12, 13756-13763. doi:10.1039/d1sc03533a

Zhan, M., Zhang, S., Huang, Z., and Xi, Z. (2015). Synthesis of α,α,α',α'-tetrachloro-Δ^1^-bipyrrolines and 4,8-dichloro-2,6-diazasemibuvallenes. *Org. Lett.* 17, 1026-1029. doi:10.1021/acs.orglett.5b00136

Zhu, X., and Chiba, S. (2016). Construction of 1-pyrroline skeletons by Lewis acid-mediated conjugate addition of vinyl azides. *Chem Commun (Camb)* 52, 2473-2476. doi:10.1039/c5cc10299e

# 8. Copies of NMR spectra for products

^1^H NMR spectrum of **3aa**

^13^C NMR spectrum of **3aa**

^1^H NMR spectrum of **3ba**

^13^C NMR spectrum of **3ba**

^1^H NMR spectrum of **3ca**

^13^C NMR spectrum of **3ca**

^1^H NMR spectrum of **3da**

^13^C NMR spectrum of **3da**

^1^H NMR spectrum of **3ea**

^19^F NMR spectrum of **3ea**

^13^C NMR spectrum of **3ea**

^1^H NMR spectrum of **3fa**

^13^C NMR spectrum of **3fa**

^1^H NMR spectrum of **3ga**

^13^C NMR spectrum of **3ga**

^1^H NMR spectrum of **3ha**

^13^C NMR spectrum of **3ha**

^1^H NMR spectrum of **3ia**

^13^C NMR spectrum of **3ia**

^1^H NMR spectrum of **3ja**

^19^F NMR spectrum of **3ja**

^13^C NMR spectrum of **3ja**

^1^H NMR spectrum of **3ka**

^13^C NMR spectrum of **3ka**

^1^H NMR spectrum of **3la**

^13^C NMR spectrum of **3la**

^1^H NMR spectrum of **3ma**

^13^C NMR spectrum of **3ma**

^1^H NMR spectrum of **3na**

^13^C NMR spectrum of **3na**

^1^H NMR spectrum of **3oa**

^19^F NMR spectrum of **3oa**

^13^C NMR spectrum of **3oa**

^1^H NMR spectrum of **3pa**

^13^C NMR spectrum of **3pa**

^1^H NMR spectrum of **3qa**

^13^C NMR spectrum of **3qa**

^1^H NMR spectrum of **3ra**

^13^C NMR spectrum of **3ra**

^1^H NMR spectrum of **3sa**

^13^C NMR spectrum of **3sa**

^1^H NMR spectrum of **3ta**

^13^C NMR spectrum of **3ta**

^1^H NMR spectrum of **3ab**


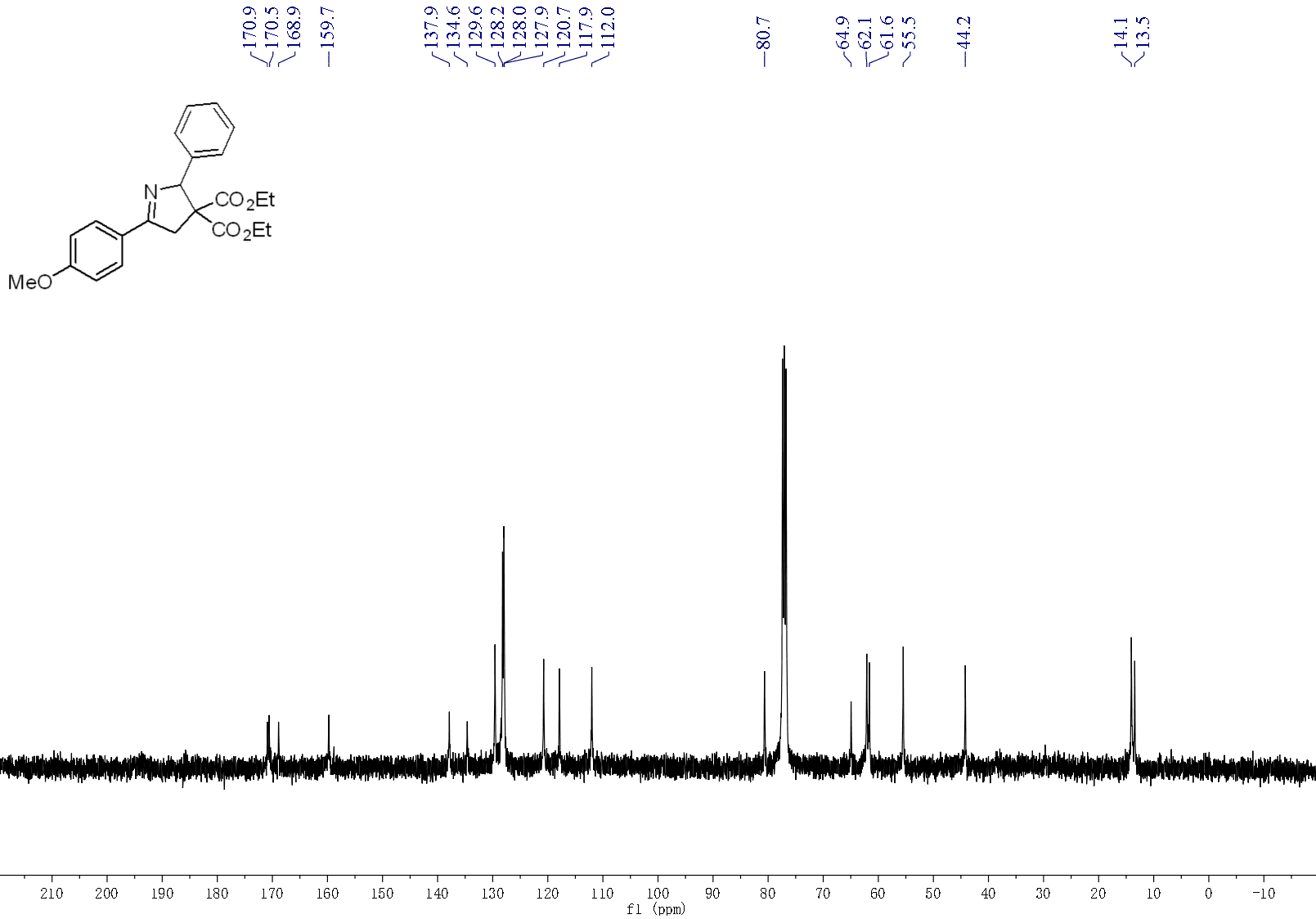


^13^C NMR spectrum of **3ab**

^1^H NMR spectrum of **3ac**

^13^C NMR spectrum of **3ac**

^1^H NMR spectrum of **3ad**

^13^C NMR spectrum of **3ad**

^1^H NMR spectrum of **3ae**

^13^C NMR spectrum of **3ae**

^1^H NMR spectrum of **3af**

^13^C NMR spectrum of **3af**

^1^H NMR spectrum of **3ag**

^19^F NMR spectrum of **3ag**

^13^C NMR spectrum of **3ag**

^1^H NMR spectrum of **3ah**

^13^C NMR spectrum of **3ah**

^1^H NMR spectrum of **3ai**

^19^F NMR spectrum of **3ai**

^13^C NMR spectrum of **3ai**

^1^H NMR spectrum of **3aj**

^13^C NMR spectrum of **3aj**

^1^H NMR spectrum of **3ak**

^19^F NMR spectrum of **3ak**

^13^C NMR spectrum of **3ak**

^1^H NMR spectrum of **3al**

^19^F NMR spectrum of **3al**

^13^C NMR spectrum of **3al**

^1^H NMR spectrum of **3am**

^13^C NMR spectrum of **3am**


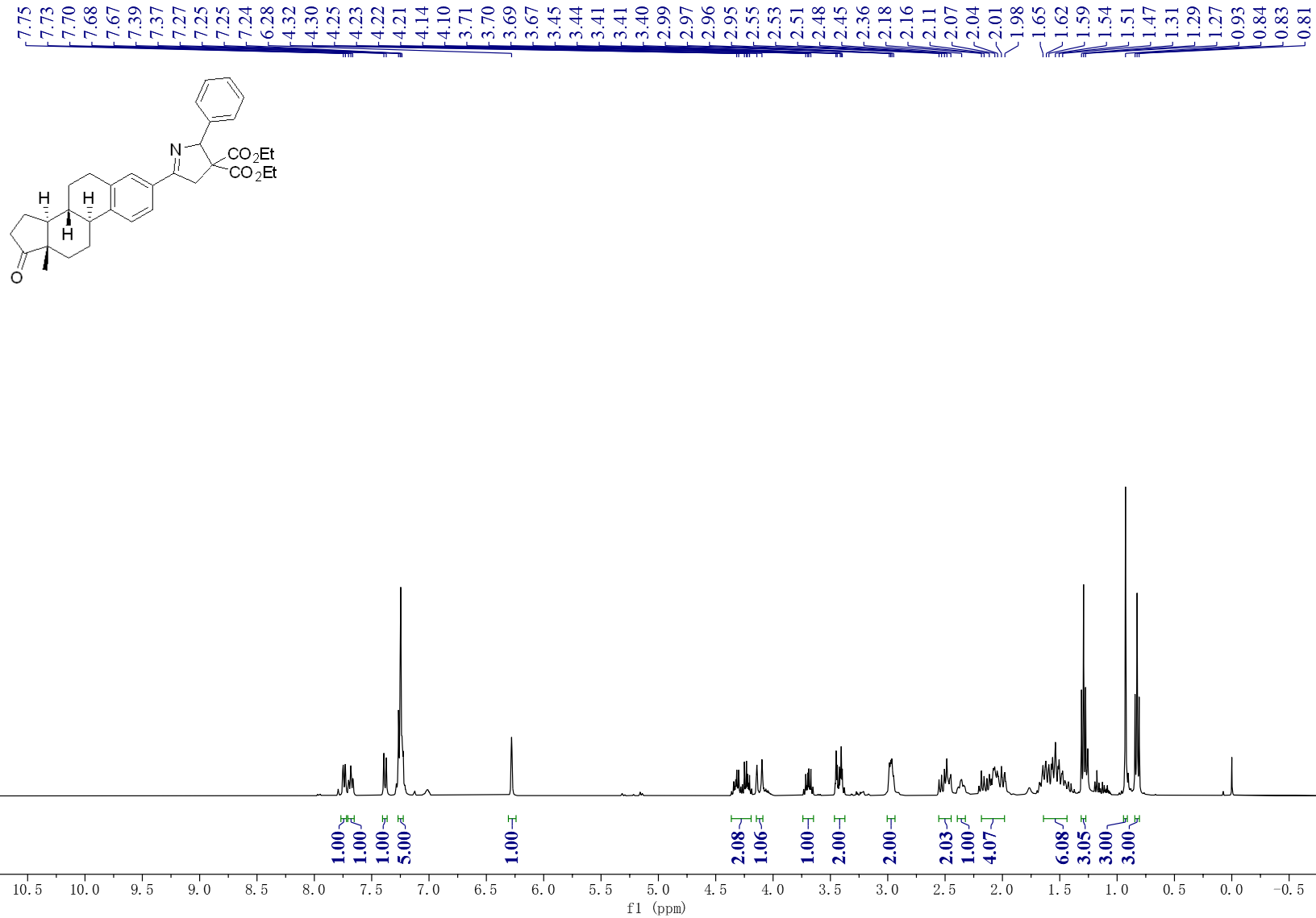


^1^H NMR spectrum of **3ap**


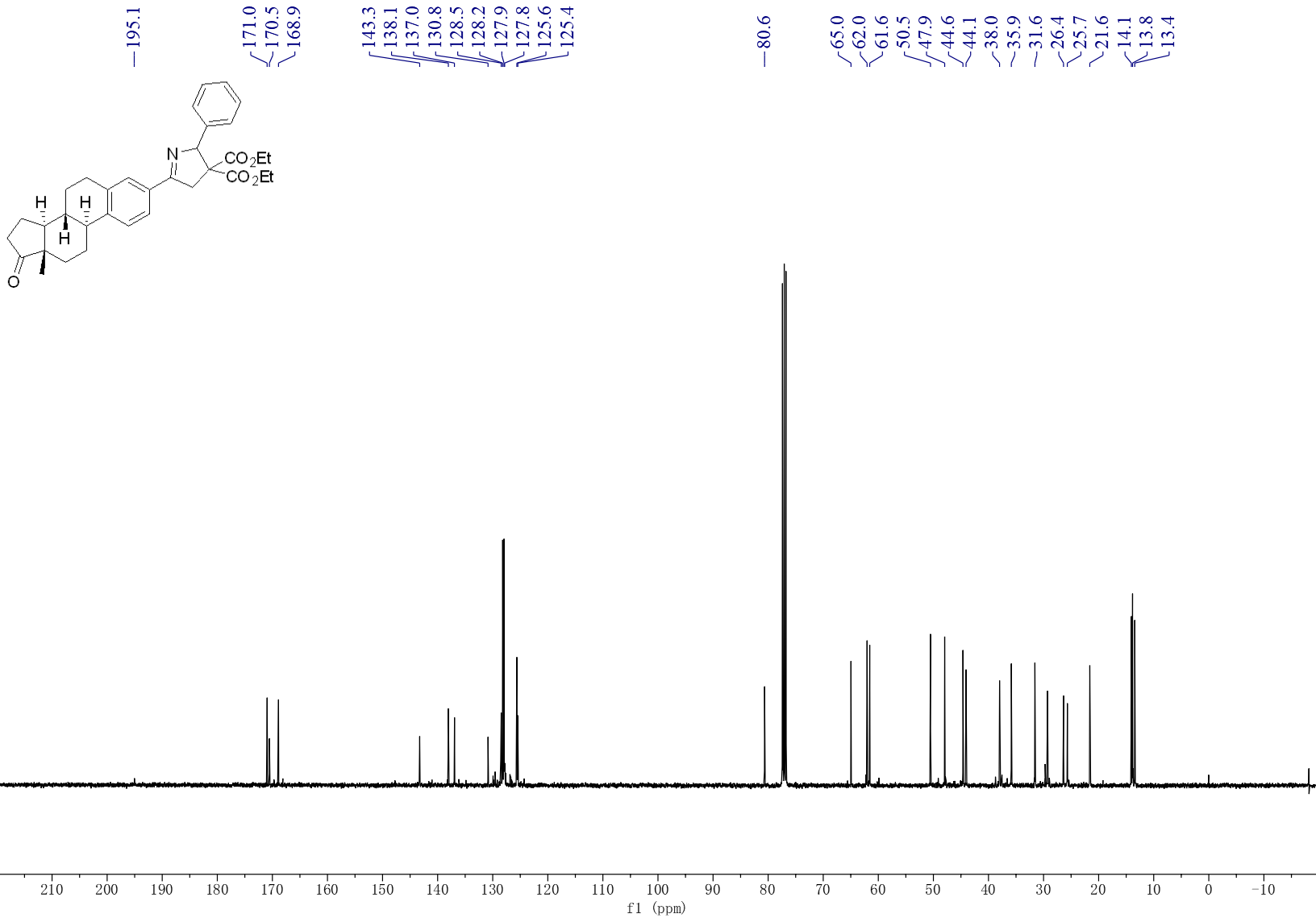


^13^C NMR spectrum of **3ap**

^1^H NMR spectrum of **4aa**

^13^C NMR spectrum of **4aa**

^1^H NMR spectrum of **4ca**

^13^C NMR spectrum of **4ca**

^1^H NMR spectrum of **4da**

^13^C NMR spectrum of **4da**

^1^H NMR spectrum of **4ea**

^19^F NMR spectrum of **4ea**

^13^C NMR spectrum of **4ea**

^1^H NMR spectrum of **4fa**

^13^C NMR spectrum of **4fa**

^1^H NMR spectrum of **4ga**

^13^C NMR spectrum of **4ga**

^1^H NMR spectrum of **4ha**

^13^C NMR spectrum of **4ha**

^1^H NMR spectrum of **4ia**

^13^C NMR spectrum of **4ia**

^1^H NMR spectrum of **4ja**

^19^F NMR spectrum of **4ja**

^13^C NMR spectrum of **4ja**

^1^H NMR spectrum of **4ka**

^13^C NMR spectrum of **4ka**

^1^H NMR spectrum of **4la**

^13^C NMR spectrum of **4la**

^1^H NMR spectrum of **4ma**

^13^C NMR spectrum of **4ma**

^1^H NMR spectrum of **4na**

^13^C NMR spectrum of **4na**

^1^H NMR spectrum of **4oa**

^19^F NMR spectrum of **4oa**

^13^C NMR spectrum of **4oa**

^1^H NMR spectrum of **4pa**

^13^C NMR spectrum of **4pa**

^1^H NMR spectrum of **4qa**

^13^C NMR spectrum of **4qa**

^1^H NMR spectrum of **4ab**

^13^C NMR spectrum of **4ab**

^1^H NMR spectrum of **4ac**

^13^C NMR spectrum of **4ac**

^1^H NMR spectrum of **4ad**

^13^C NMR spectrum of **4ad**

^1^H NMR spectrum of **4ae**

^13^C NMR spectrum of **4ae**

^1^H NMR spectrum of **4af**

^13^C NMR spectrum of **4af**

^1^H NMR spectrum of **4ag**

^19^F NMR spectrum of **4ag**

^13^C NMR spectrum of **4ag**

^1^H NMR spectrum of **4ah**

^13^C NMR spectrum of **4ah**

^1^H NMR spectrum of **4aj** and **4aj'**

^13^C NMR spectrum of **4aj** and **4aj'**

^1^H NMR spectrum of **4an**

^13^C NMR spectrum of **4an**

^1^H NMR spectrum of **4ao**

^13^C NMR spectrum of **4ao**

^1^H NMR spectrum of **5**

^19^F NMR spectrum of **5**

^13^C NMR spectrum of **5**

^1^H NMR spectrum of **6**

^13^C NMR spectrum of **6**

^1^H NMR spectrum of **7**

^13^C NMR spectrum of **7**

^1^H NMR spectrum of **8**

^13^C NMR spectrum of **8**

^1^H NMR spectrum of **9**

^13^C NMR spectrum of **9**

^1^H NMR spectrum of **3aa'**

^13^C NMR spectrum of **3aa'**

^1^H NMR spectrum of **2a'**

^13^C NMR spectrum of **2a'**
