# Supplementary material for: Chemodivergent photocatalytic access to 1-pyrrolines and 1-tetralones involving switchable C(sp3)–H functionalization
Source: Front Chem. 2022 Oct 25;10:1058596. doi: 10.3389/fchem.2022.1058596 (PMC9641198; doi:10.3389/fchem.2022.1058596)

checkCIF/PLATON report

Structure factors have been supplied for datablock(s) XDX9_0m

THIS REPORT IS FOR GUIDANCE ONLY. IF USED AS PART OF A REVIEW PROCEDURE FOR PUBLICATION, IT SHOULD NOT REPLACE THE EXPERTISE OF AN EXPERIENCED CRYSTALLOGRAPHIC REFEREE.

No syntax errors found. CIF dictionary Interpreting this report

Datablock: XDX9_0m

Bond precision: C-C = 0.0026 A Wavelength=0.71073

Cell:	a=8.776(2)	b=10.530(3)	c=13.052(3)
	alpha=71.261(3)	beta=76.132(3)	gamma=81.031(3)
Temperature:	296 K		

	Calculated	Reported
Volume	1104.8(5)	1104.9(5)
Space group	P -1	P -1
Hall group	-P 1	-P 1
Moiety formula	C24 H27 N O5	?
Sum formula	C24 H27 N O5	C24 H27 N O5
Mr	409.47	409.46
Dx,g cm-3	1.231	1.231
Z	2	2
Mu (mm-1)	0.086	0.086
F000	436.0	436.0
F000'	436.22	
h,k,lmax	11,13,16	11,13,16
Nref	5079	4993
Tmin,Tmax	0.982,0.985	
Tmin'	0.982	

Correction method= Not given

Data completeness= 0.983 Theta(max)= 27.507

R(reflections)= 0.0560(3855) wR2(reflections)= 0.2031(4993)

S = 1.171 Npar= 275

The following ALERTS were generated. Each ALERT has the format
test-name_ALERT_alert-type_alert-level.
Click on the hyperlinks for more details of the test.

Alert level C

```
PLAT052_ALERT_1_C Info on Absorption Correction Method   Not Given     Please Do !
PLAT220_ALERT_2_C NonSolvent   Resd 1  C   Ueq(max)/Ueq(min) Range        3.7 Ratio
PLAT222_ALERT_3_C NonSolvent Resd 1  H   Uiso(max)/Uiso(min) Range        4.3 Ratio
PLAT360_ALERT_2_C Short  C(sp3)-C(sp3) Bond  C20      - C21      .       1.41 Ang.
PLAT906_ALERT_3_C Large K Value in the Analysis of Variance ......      2.091 Check
PLAT911_ALERT_3_C Missing FCF Refl Between Thmin & STh/L=    0.600         28 Report
```

Alert level G

```
PLAT154_ALERT_1_G The s.u.'s on the Cell Angles are Equal ..(Note)      0.003 Degree
PLAT793_ALERT_4_G Model has Chirality at C8          (Centro SPGR)          R Verify
PLAT883_ALERT_1_G No Info/Value for _atom_sites_solution_primary .     Please Do !
PLAT910_ALERT_3_G Missing # of FCF Reflection(s) Below Theta(Min).          1 Note
PLAT912_ALERT_4_G Missing # of FCF Reflections Above STh/L=  0.600         58 Note
PLAT933_ALERT_2_G Number of HKL-OMIT Records in Embedded .res File         11 Note
PLAT941_ALERT_3_G Average HKL Measurement Multiplicity ..........         2.1 Low
PLAT965_ALERT_2_G The SHELXL WEIGHT Optimisation has not Converged     Please Check
PLAT978_ALERT_2_G Number C-C Bonds with Positive Residual Density.          8 Info
PLAT992_ALERT_5_G Repd & Actual _reflns_number_gt Values Differ by          5 Check
```

0 **ALERT level A** = Most likely a serious problem - resolve or explain
0 **ALERT level B** = A potentially serious problem, consider carefully
6 **ALERT level C** = Check. Ensure it is not caused by an omission or oversight
10 **ALERT level G** = General information/check it is not something unexpected

3 ALERT type 1 CIF construction/syntax error, inconsistent or missing data
5 ALERT type 2 Indicator that the structure model may be wrong or deficient
5 ALERT type 3 Indicator that the structure quality may be low
2 ALERT type 4 Improvement, methodology, query or suggestion
1 ALERT type 5 Informative message, check

It is advisable to attempt to resolve as many as possible of the alerts in all categories. Often the minor alerts point to easily fixed oversights, errors and omissions in your CIF or refinement strategy, so attention to these fine details can be worthwhile. In order to resolve some of the more serious problems it may be necessary to carry out additional measurements or structure refinements. However, the purpose of your study may justify the reported deviations and the more serious of these should normally be commented upon in the discussion or experimental section of a paper or in the "special_details" fields of the CIF. checkCIF was carefully designed to identify outliers and unusual parameters, but every test has its limitations and alerts that are not important in a particular case may appear. Conversely, the absence of alerts does not guarantee there are no aspects of the results needing attention. It is up to the individual to critically assess their own results and, if necessary, seek expert advice.

Publication of your CIF in IUCr journals

A basic structural check has been run on your CIF. These basic checks will be run on all CIFs submitted for publication in IUCr journals (*Acta Crystallographica*, *Journal of Applied Crystallography*, *Journal of Synchrotron Radiation*); however, if you intend to submit to *Acta Crystallographica Section C* or *E* or *IUCrData*, you should make sure that full publication checks are run on the final version of your CIF prior to submission.

Publication of your CIF in other journals

Please refer to the *Notes for Authors* of the relevant journal for any special instructions relating to CIF submission.

PLATON version of 18/12/2021; check.def file version of 18/12/2021

Datablock XDX9_0m - ellipsoid plot

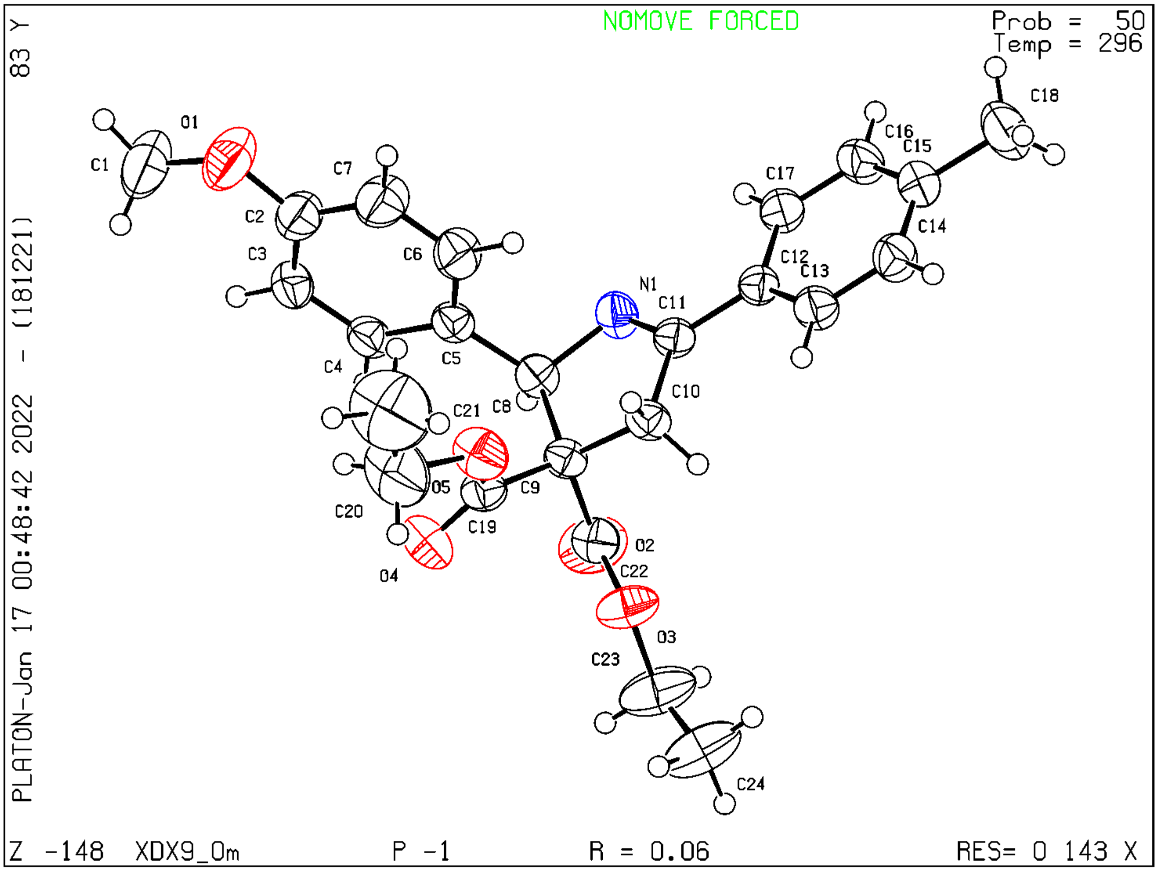

Supplement: Supplementary file 3 [file DataSheet1.PDF]
